# Effects of specific disease mutations in non-muscle myosin 2A on its structure and function

**DOI:** 10.1016/j.jbc.2023.105514

**Published:** 2023-11-30

**Authors:** David Casas-Mao, Glenn Carrington, Marta Giralt Pujol, Michelle Peckham

**Affiliations:** Astbury Centre for Structural Molecular Biology & School of Molecular and Cellular Biology, Faculty of Biological Sciences, University of Leeds, Leeds, UK

**Keywords:** myosin, electron microscopy (EM), circular dichroism (CD), fluorescence recovery after photobleaching (FRAP), cytoskeleton, non-muscle myosin 2A, MYH9 disease

## Abstract

Non-muscle myosin 2A (NM2A), a widely expressed class 2 myosin, is important for organizing actin filaments in cells. It cycles between a compact inactive 10S state in which its regulatory light chain (RLC) is dephosphorylated and a filamentous state in which the myosin heads interact with actin, and the RLC is phosphorylated. Over 170 missense mutations in MYH9, the gene that encodes the NM2A heavy chain, have been described. These cause MYH9 disease, an autosomal-dominant disorder that leads to bleeding disorders, kidney disease, cataracts, and deafness. Approximately two-thirds of these mutations occur in the coiled-coil tail. These mutations could destabilize the 10S state and/or disrupt filament formation or both. To test this, we determined the effects of six specific mutations using multiple approaches, including circular dichroism to detect changes in secondary structure, negative stain electron microscopy to analyze 10S and filament formation *in vitro*, and imaging of GFP-NM2A in fixed and live cells to determine filament assembly and dynamics. Two mutations in D1424 (D1424G and D1424N) and V1516M strongly decrease 10S stability and have limited effects on filament formation *in vitro*. In contrast, mutations in D1447 and E1841K, decrease 10S stability less strongly but increase filament lengths *in vitro*. The dynamic behavior of all mutants was altered in cells. Thus, the positions of mutated residues and their roles in filament formation and 10S stabilization are key to understanding their contributions to NM2A in disease.

Non-muscle myosin 2A (NM2A), encoded by the MYH11 gene, is one of three non-muscle class 2 myosin isoforms expressed in mammals (1). In common with other class 2 myosins, the heavy chain consists of a motor domain, followed by a light chain binding domain comprised of two IQ domains (an uninterrupted amphiphilic seven-turn α-helix so called based on the first two amino acids, isoleucine and glutamine, in the sequence), and a coiled-coil tail that dimerizes the two heavy chains and enables them to assemble into filaments. Two types of light chains bind to the IQ motifs. The essential light chain (MYL6) binds to the first IQ, and the regulatory light chain (MYL9) binds to the second. Individual molecules of NM2A form a compact shutdown ‘10S’ state (2, 3) in which the coiled-coil tail wraps around the motor domains, and the ATPase activity is very low. The coiled-coil interacts with the motor domain of the blocked head in this shutdown state, and with the N-terminal SH3 domain. Phosphorylation of the regulatory light chain (RLC) on serine 19 (4) disrupts the shutdown state and enables filament formation (Fig. 1, *A* and *B*). Recent Cryo-EM structures of smooth muscle myosin, which fold up in a similar way, have helped to explain how phosphorylation disrupts this state 5, 6, 7.

**Figure 1:**
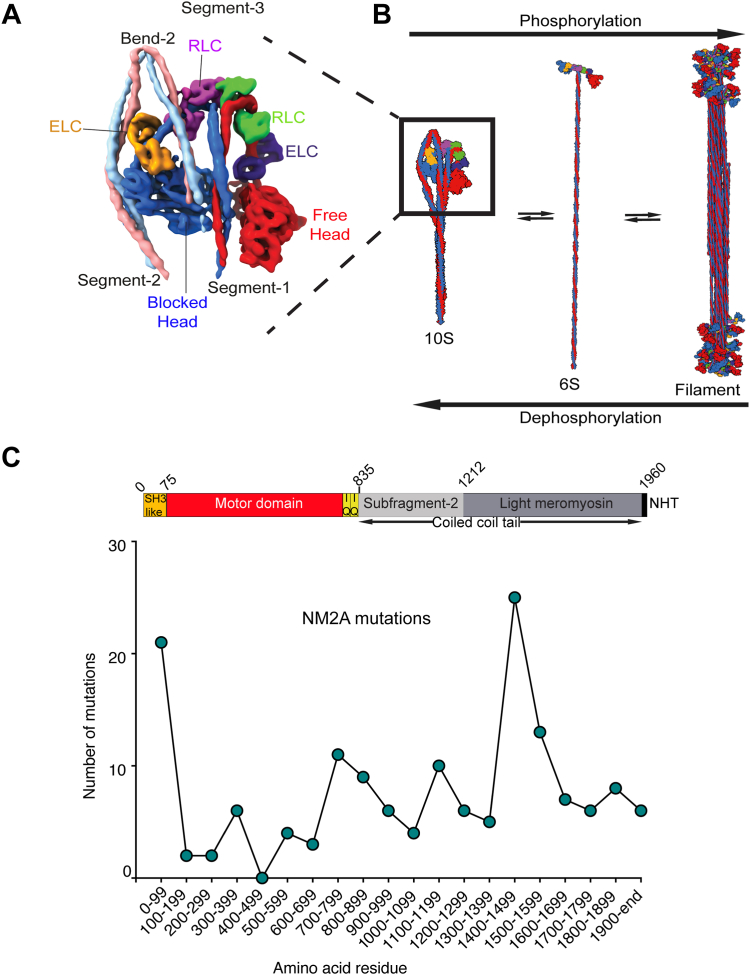
**NM2A shutdown and filamentous states and positions of mutations.***A*, overview of the head region of 10S shutdown myosin, constructed from the split density map for smooth muscle myosin (6), which folds up in a similar way to NM2A. The two α helices that make up the coiled-coil from each of the two chains of the myosin dimer are shown colored *pink* (from the free head) and *light blue* (from the blocked head). The motor domain of the blocked head is colored *blue* and that of the free head is colored *red*. The essential light chains (ELCs) are colored *orange* (blocked head) and *dark blue* (free head). The regulatory light chains (RLCs) are colored *purple* (blocked head) and *green* (free head). *B*, phosphorylation of the RLCs results in an extended 6S molecule that is competent to form filaments. *C*, analysis of the frequency of mutations in NM2A. The number of missense mutations in each block of 100 residues is shown. A linear representation of the amino acid sequence of human NM2A is shown above the graph to indicate in which part of the molecule these mutations lie.

The coiled-coil tail comprises a proximal (subfragment-2 (S2)) and distal region (light meromyosin (LMM)), and it is LMM that is important for filament assembly (Fig. 1*C*). Individual helices in the coiled-coil follow a 7-residue repeat (heptad: *abcdefg*). Amino acid residues in the *a* and *d* positions are typically hydrophobic in nature (8). Residues in the *g* and *e* positions are often charged and can form ionic interactions (i, i-5) to stabilize the coiled-coil. Residues in the *b*, *c*, and *f positions* are located on the outside of the helix and are thought to be involved in filament assembly. Non-muscle and smooth muscle myosin heavy chains additionally contain three skip residues, in which an extra single residue interrupts the canonical heptad pattern, locally disrupting the coiled-coil (9). At the end of the coiled-coil is a non-helical tailpiece (NHT), 34 residues in length. Myosin molecules are assembled into filaments based on charge, with LMM containing alternating portions of positive and negative charge, followed by the largely positively charged non-helical tailpiece (10). Phosphorylation of Ser1943 in the NHT and phosphorylation of Ser1916, just upstream in the coiled-coil, both inhibit filament assembly (reviewed in (11)).

Mutations in the MYH9 gene that encodes NM2A cause MYH9-related disease, an autosomal dominant disease that affects approximately one in 312,000 people (12, 13). While most cells express at least two types of non-muscle myosin two isoforms, platelets only express NM2A (14). Platelets change their shape when activated, and this is driven by actin filament assembly and activation of NM2A following phosphorylation of its RLC followed by filament assembly (Fig. 1*B*) (15, 16). As NM2A is the only isoform of non-muscle myosin in platelets, it is not surprising that MYH9 mutations cause bleeding disorders. However, MYH9 mutations also lead to kidney defects, cataracts, and/or deafness, collectively known as MYH9 disease (11, 17). It is still not clear exactly how these mutations lead to disease. Current ideas include effects on the stability of the shutdown state that mean incorrect regulation of the switch between shutdown and active, filamentous states, a propensity for the mutant isoforms to aggregate, and effects on contractility depending on the position of the mutation (17).

Recent work has attempted to understand how specific mutations affect the behavior of NM2A, using a range of approaches, including animal models, human samples, and *in vitro* studies. In human platelets, mutations in the motor domain were recently shown to increase the association of NM2A with the actin cytoskeleton at the membrane of red blood cells, likely due to effects on motor activity, extending the phenotype of these mutations (18). A range of effects was observed using mouse models combined with *in vitro* experiments for specific mutations in the motor domain (R702C) and in the coiled-coil (D1424N and E1841K) including altered chemotaxis of megakaryocytes in the bone marrow, reduced spreading of platelets on fibrinogen and an over-assembly of E1841K into filaments *in vitro* (wider and longer filaments) but not in cells. Mutations have also been shown to affect secondary structure and paracrystal formation by LMM (19). However, it is not clear if any of these or other mutations affect the stability of the shutdown state.

In this study, we first built a homology model of NM2A based on our recent structure of shutdown SMM (6), to better understand how mutations in the coiled-coil might interfere with the role of NM2A in forming a shutdown state. We used this model to select five specific disease mutations in three different residues in a region of the coiled-coil that wraps around the motor domain to form the shutdown state D1424 (D1424G and D1424N), D1447 (D1447G and D1447H), and V1516 (V1615M) for further experimental work. In addition, we chose a sixth mutation in a region of coiled-coil near the end of the tail (E1841K) that does not interact with the myosin heads. We tested the effects of these mutations using expressed and purified proteins *in vitro*, and using GFP-tagged proteins expressed in an epithelial cell line using a comprehensive range of experimental approaches. This enabled us to determine the effect of these mutations on secondary structure and filament formation, as well as on the formation of the shutdown state *in vitro* and filament formation in cells to gain new insight into the effects of these mutations on NM2A.

## Results

### Mutational analysis and predicting potential effects of mutations using an NM2A homology model

Over 220 disease-causing mutations have been reported for the gene that encodes NM2A (MYH9: accessed Jan 2023, Human Genome Mapping Database: https://www.hgmd.cf.ac.uk/ac/index.php, and see Fig. S1 and Table 1). Of these over 170 are missense or nonsense, with most missense (single amino acid substitutions). The most common mutation in the motor domain is in residue R702, which is found in the SH1 helix in the motor domain and results in severe disease (11). However, an analysis of the positions of all the reported mutations shows that they are found throughout the amino acid sequence of NM2A, with roughly two-thirds located in the coiled-coil tail (6) (Figs. 1*C*; S1 and Table 1). There are 15 different missense mutations in the N-terminal region of NM2A, which is folded into an SH3-like domain that interacts with the coiled-coil in the shutdown molecule, evidencing the importance of this region. Missense mutations are also particularly frequent in the region of the coiled-coil that interacts with the motor domain of the blocked head to stabilize the shutdown state (between residues 1400 and 1600) (6). This region of the coiled-coil (LMM: light meromyosin) is also important for the aggregation of molecules into filaments. This raises the possibility that mutations in this region can either interfere with the stability of the shutdown 10S state, interfere with filament formation, or both. Of the mutations found between residues 1400 to 1600, 5 separate disease-causing mutations have been reported for D1424 (D1424N/H/Y/E/G), four for D1447 (D1447G/H/Y/V), and two for V1516 (V1516L/M) (Table S1). This prompted us to choose mutations in these residues for further study, together with an additional mutation in the coiled-coil not in this region (E1841K).

**Table 1:** Summary of the effects of mutations studied

The % changes compared to WT are shown for each of the parameters. Gray highlight indicates decreases and bold text indicates changes that were statistically significant, as shown on the individual figures.

**Figure 2:**
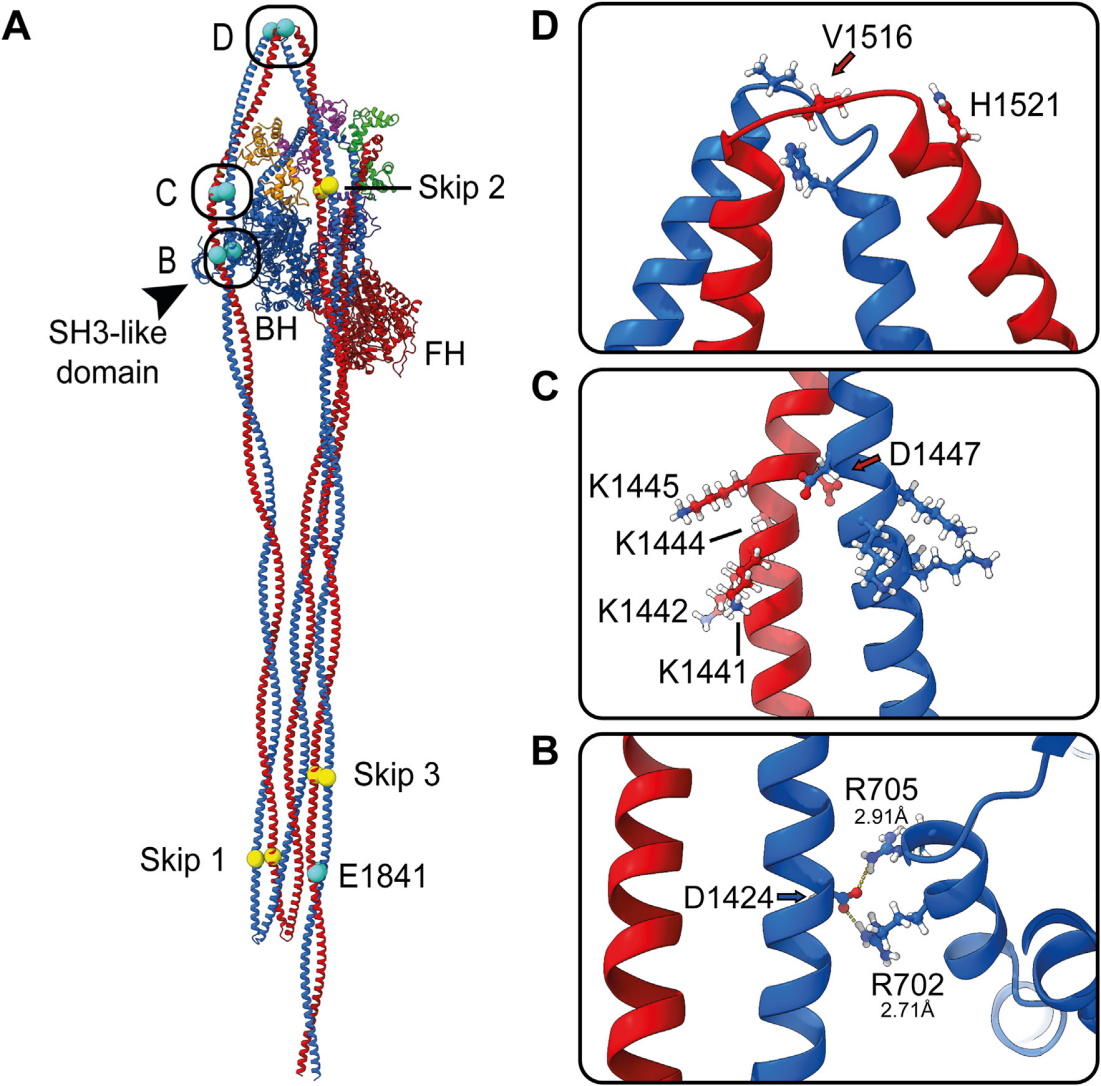
**NM2A homology model.***A*, the full-length homology model. The positions of the skip residues (*yellow*) and residues E1841, D1424, D1447, and V1516 (*cyan*) are highlighted. The position of the N-terminal SH3-like domain is also indicated. *B*–*D*, boxed regions are shown enlarged on the *right-hand side*. *B*, the potential salt bridge formation between D1424 in the coiled-coil with R702 and/or R705 in the converter domain of the blocked head. Inter-atomic distances between hydroxyl and guanidium groups of D and R groups are indicated under the residue number. *C*, the position of D1447 in a region of coiled-coil that does not appear to interact with the blocked head. This residue is upstream of a patch of basic residues as indicated. *D*, the position of V1516 at bend 2 together with the position of H1521 downstream. BH, blocked head; FH, free head.

To explore the potential roles of D1424, D1447, and V1516 in stabilizing the shutdown state, we generated a homology model of shutdown NM2A based on the structure for shutdown SMM (6) and mapped their positions onto the structure (Fig. 2*D*). This suggests that D1424 is located in a region of the coiled-coil that could interact with residues R702 and R705 in the motor domain in the blocked head by forming a salt bridge, helping to stabilize the shutdown state (Fig. 2). Mutations in this residue might thus be expected to destabilize the formation of the shutdown 10S state, by removing this interaction. D1447 is found in a region that does not appear to directly interact with the blocked head and thus might not be expected to have a strong effect of the formation of the 10S state. V1516 is very close to the bend (bend-2) between segment-2 and segment-3 of the coiled-coil (Fig. 2). Mutations here could also destabilize the formation of the shutdown state. Mapping E1841 onto the homology model shows that it is close to the C-terminal tail. The charge change to lysine (K) in this residue could destabilize the shutdown state if it is involved in stabilizing interactions between the three coiled-coil segments. Finally, all of the mutations have the potential to affect filament formation once the molecules become extended and assemble into bipolar filaments. D1424 is in a *c* position and D1447 and E1841 are both in an *e* position in the heptad repeat. These residues are found toward the outside of the coiled-coil and could contribute to its overall net charge. A change in charge could disrupt filament formation. V1516M is in a *d* position, which is normally involved in forming the hydrophobic seam of the coiled-coil. To test these ideas, we performed a set of experiments to test the effects of the mutation on secondary structure, filament formation and 10S formation.

### Circular dichroism of LMM constructs identifies mutations that affect helicity and thermal stability

**Figure 3:**
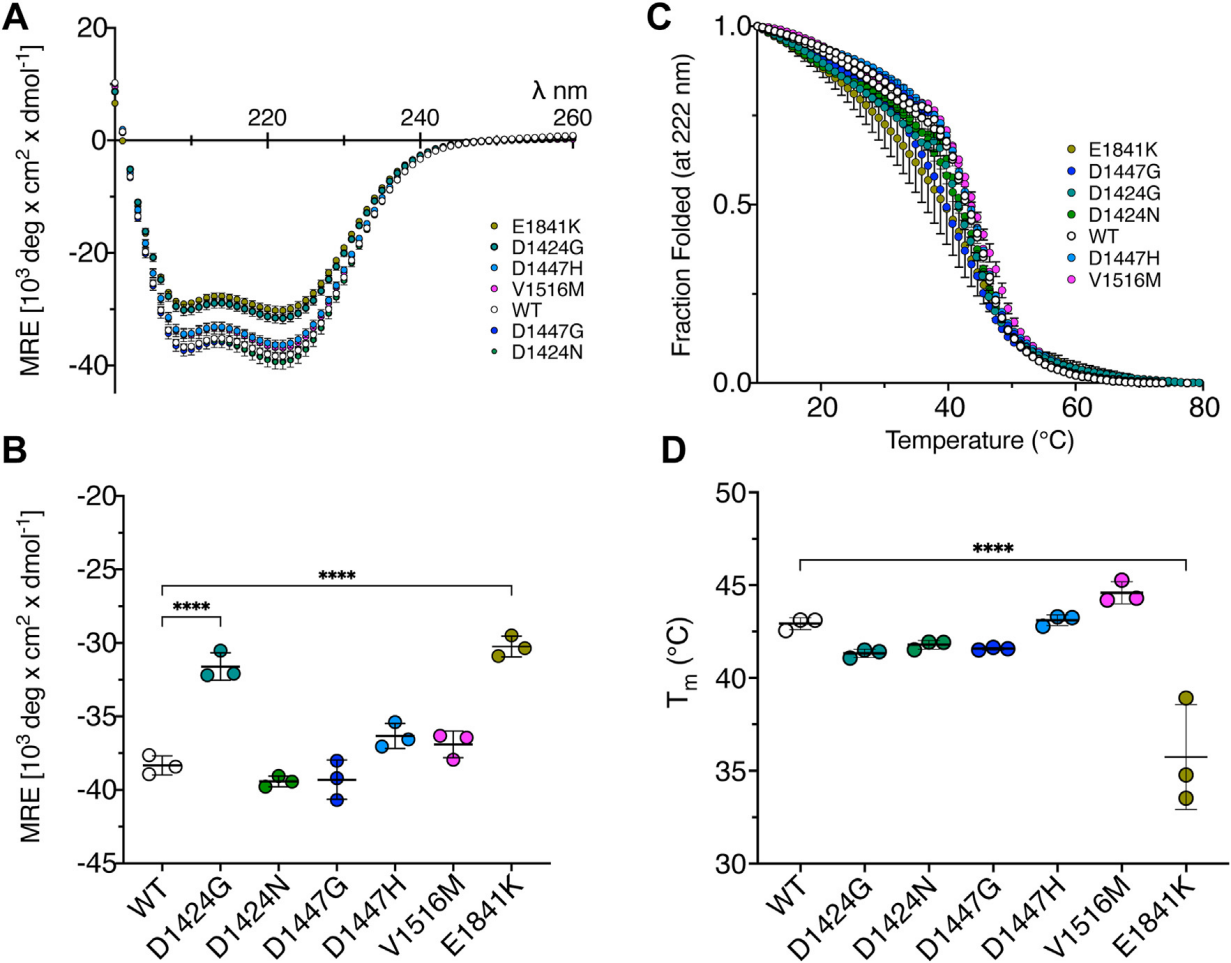
**Secondary structure of wild type and mutant LMM constructs.***A*, CD spectra for WT and mutant LMM constructs in 100 mM NaCl, 10 mM sodium phosphate buffer (pH 7.4) at 10 °C. *B*, the mean residue ellipticity (MRE) values were measured at 222 nm for WT and mutant LMM constructs at 10 °C. *C*, thermal melting curves for WT and mutant constructs spanning a temperature range of 10 to 80 °C. *D*, Tm for each of the constructs. Plots additionally show the average values for three separate measurements, together with the SD in *C* and *D*. Values were compared by a 1-way ANOVA, in which each of the values were compared to wild type, using a posthoc Dunnett test. Significant differences between values are indicated on the plots. ∗∗∗∗*p* < 0.0001. Measurements from three separate experiments for each construct.

Circular dichroism (CD) of expressed and purified full-length LMM constructs (748 residues, residues 1212–1960 of the heavy chain sequence for human NM2A) showed that all of the constructs were α-helical, with mean residue ellipticity (MRE) minima at wavelengths of 208 and 222 nm (Fig. 3*A*). Two of the six mutant constructs, D1424G and E1814K, significantly decreased the helical content of LMM compared to WT, as evidenced by the increased MRE values at 222 nm (Fig. 3, *A* and *B*). D1447H and V1516M slightly decreased helicity, but this difference was not significant. D1424N and D1447G did not affect helicity.

Thermal denaturation experiments show that four of the mutants, D1424G, D1424N, D1447G, and E1841K, were less thermally stable than WT, as evidenced by a decrease in the temperature at which half the helix has melted (T_m_) (Fig. 3, *C* and *D*). However, this reduction was only significant for E1841K. The mutant V1615M showed a small increase in T_m_ but this effect was not significantly different from WT (Fig. 3, *C* and *D*). Of all the mutations, E1814K is clearly the most destabilizing as it shows the largest reduction in the MRE value at 222 nm and in thermal stability, with D1424G the next most destabilizing. Interestingly, a previous report using a longer coiled-coil construct (residues 1102–1960) found that while E1841K affected coiled-coil stability, it has no effect on thermal stability (19). However, longer regions of coiled-coil can more effectively mask small changes in helicity and thermal stability arising from a single point mutation (20).

### Negative stain EM reveals varied effects on filament formation *in vitro*

**Figure 4:**
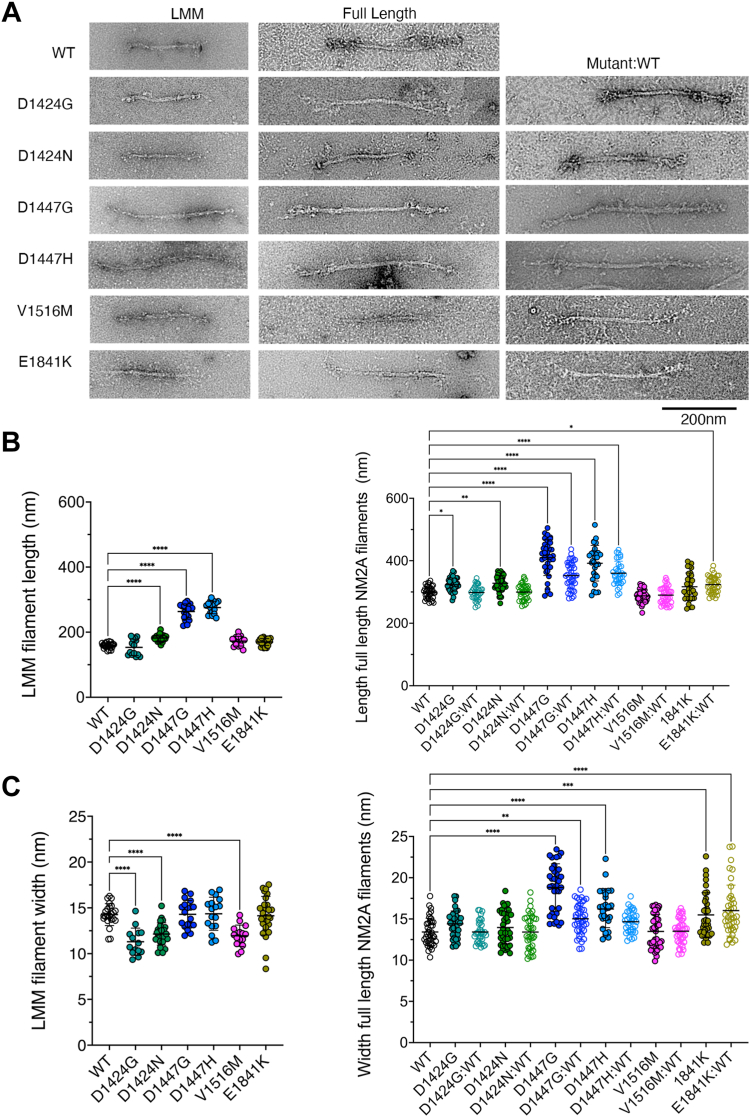
**Effects of mutations on filament length *in vitro*.***A*, representative negative stain images of filaments formed either by GST-LMM or by full-length NM2A constructs for WT and each of the mutant constructs. Additional images for mixed filaments (50:50 WT:mutant) are also shown. Scale Bar as shown. *B*, filament length measurements for GST-LMM and full-length NM2A filaments. Equivalent width measurements are shown in (*C*). Mean ± SD values are additionally shown on the plots. Images were analyzed using FIJI. Length and width data were compared by a 1-way ANOVA, in which each of the values were compared to wild type, using a post hoc Dunnett test. Significant differences between mutants and WT are indicated. ∗*p* < 0.05, ∗∗*p* < 0.01, ∗∗∗*p* < 0.001, ∗∗∗*p*< 0.0001. Measurements from three separate repeats of nsEM grids for each construct. N varies from 14 to 29 for GST-LMM measurements, from 30 to 42 for full-length filaments (homogeneous and 50:50 mix).

Negative stain electron microscopy (nsEM) images were collected to determine the effects of each mutation on its ability to assemble into filaments. We used both GST-LMM and full-length (FL) constructs in these experiments. The Lgion of LMM prevents parafilament formation and enables small mini-filaments to form as reported previously (21) (Fig. 4*A*). The average length of WT GST-LMM mini-filaments was 158.7 ± 8.1 nm (mean ± SD) and the width was 14.3 ± 1.2 nm (Fig. 4*B*). The average length of WT NM2A FL-filaments was 298.2 ± 19.5 nm (mean ± SD), and their width was 13.4 ± 1.7 nm (Fig. 4, *A* and *B*, right). These dimensions are consistent with previously reported dimensions of FL filaments with a length and width of 301 ± 24 nm and 11.2 ± 2.4 nm, respectively (22). The shorter length measured for the GST-LMM minifilaments can be attributed to their lack of S-2 and myosin heads.

All the missense mutations affected filament formation. The two missense mutations in D1447 (D1447G and D1447H) had the largest effects. These two mutations significantly increased filament length compared to WT in both GST-LMM, and filaments formed by full-length NM2A (full-length filaments). There was additionally an increase in filament width in FL filaments. Mutations in D1424 (D1424G and D1424N) had more minor effects. GST-LMM filament lengths were similar to the wild type but significantly narrower. FL filaments were significantly longer, but the increase in length was smaller compared to mutations in D1447, and their width was unchanged. This result is broadly similar to earlier reports, which found that D1424N had no significant effect on filament length or width *in vitro* (23) and effects on paracrystal formation was relatively minor (19). The mutation V1516M had no effect on filament length for either GST-LMM or FL filaments. Although width was slightly decreased in GST LMM filaments, there was no effect for full-length filaments. Despite the large destabilizing effect on secondary structure that we observed for E1841K it had a relatively small effect on filament formation apart from a significant increase in width in full-length filaments. This is somewhat similar to an earlier report that filament width increased for E1841K, but also found filament length increased (23). However, paracrystals of E1841K were different in morphology from those of the wild type (19).

As mutations in humans are autosomal dominant, we additionally assayed the effects of mutations in filaments formed by a 50:50 mixture of wild-type (WT) and mutant FL NM2A. While we cannot be completely confident that the filaments do comprise a 50:50 mix, if the mutant protein fails to co-assemble with the WT protein, then we should expect to see two populations of filaments representing WT and the mutant. Likewise, if the mutant NM2A strongly affects filament formation we might expect not to see filaments at all.

For both mutations in D1424, filament lengths for mixtures of 50:50 WT:mutant were no longer increased compared to WT. However, for mutations in D1447, the overall filament length was still significantly increased. Similarly, filament widths were still increased for both mutations in D1447, although this was only significant for D1447G. No effect was seen for V1516M. For E1841K, filament widths remained increased compared to WT, and filament length was now significantly increased. Overall, the effects of the mutations on filament formation *in vitro* appear to depend on the position of the residue mutated, but there are clear changes to filament packing *in vitro* for the majority of the mutations.

### Effects of mutations on GFP-NM2A filament lengths in cells using STED microscopy

**Figure 5:**
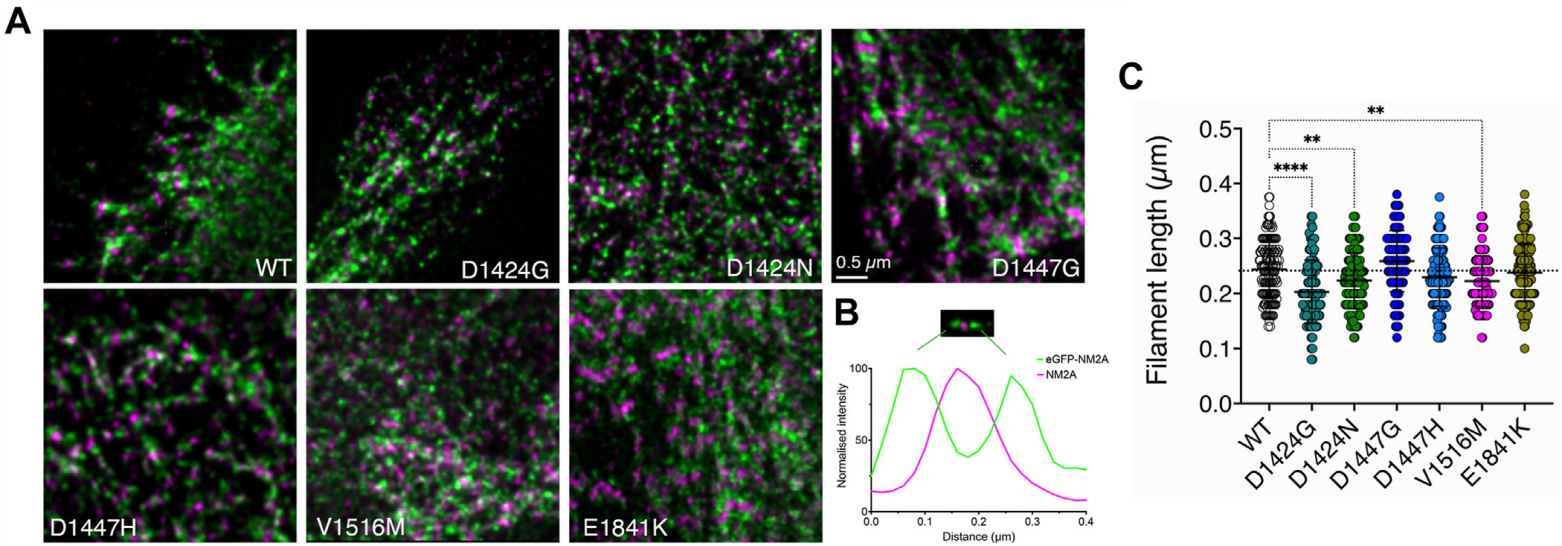
**Filament lengths of GFP-NM2A filaments in cells.***A*, representative 2D STED images for WT and mutant NM2A (*green*), in which GFP is fused to the N-terminal motor domain, and thus labels each end of these bipolar filaments. Cells were co-stained for NM2A using an antibody that recognizes the C-terminal domain and labels the central region of the filament. Images were obtained using 2D-STED imaging, which has a nominal resolution of ∼50 nm in XY. Filament lengths were analyzed from peak-peak distances across a filament as shown in (*B*) where the two ends of the filament marked by eGFP surround the central region, marked by the antibody (in *magenta*). *C*, summary of individual measurements from multiple cells for at least two biological replicates shown superimposed on box and whisker plots. Values were compared by a 1-way ANOVA, in which each of the values were compared to wild type, using a post hoc Dunnett test*.* Statistically significant differences are denoted as follows: ∗∗∗∗*p* < 0.001; ∗∗*p* < 0.01. The *dotted line* indicates the mean value for WT. Measurements from three separate experiments. (WT: n = 141, D1424G: n = 100, D1424N: n = 156, D1447G: n = 107, D1447H: n = 138, V1516M: n = 132, E1841K: n = 170).

Next, we assessed the potential impact of the mutants on filament formation in cells, using GFP-NM2A and STED imaging, which has a resolution of approximately 50 nm (Fig. 5*A*). In this instance, as the BPH1 cells used in the experiments also express endogenous NM2A, we expect that the filaments formed in cells will comprise both WT and mutant NM2A molecules. We were careful to select cells in which expression levels of GFP-NM2A were similar across all of the cells for WT and mutant constructs to enable comparison between WT and mutant constructs and excluded cells that highly overexpressed GFP-NM2A. However, we do not know if there is a true 50:50 WT:mutant ratio. Filaments were stained for eGFP, which marks the position of the myosin heads at the ends of the filaments, and for NM2A in the central region of the filaments using an antibody that recognizes the C-terminal region of NM2A (Fig. 5, *A* and *B*). In contrast to the results *in vitro*, D1424G and D1424N both significantly decreased filament length, as did V1516M (Fig. 5*C*), while filament lengths measured for the two mutations in D1447 and mutations in E1841K were similar to those for WT.

### Effects of mutations on NM2A dynamics in live cells using FRAP (fluorescence recovery after photobleaching)

**Figure 6:**
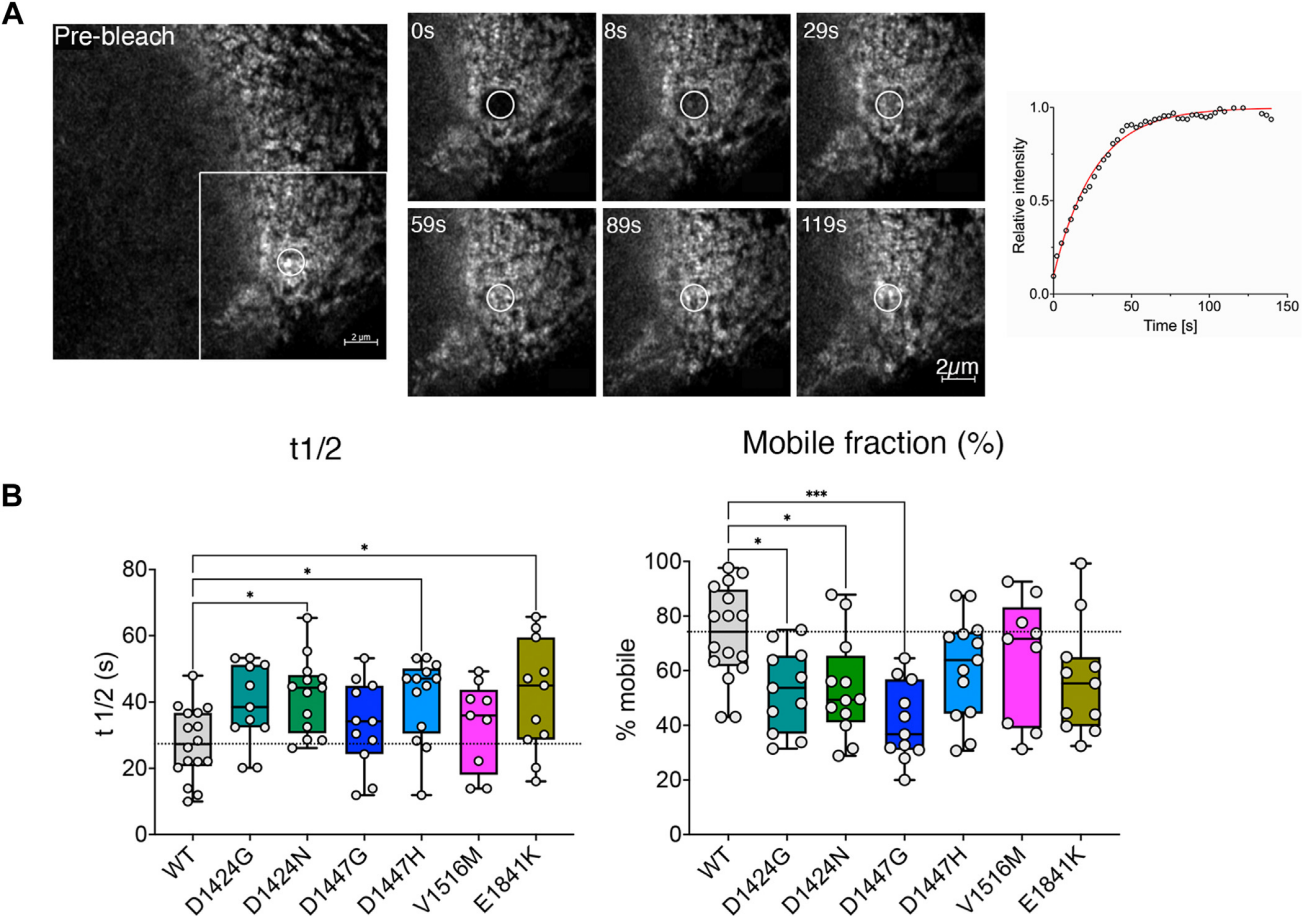
**FRAP of GFP-NM2A filaments in cells.***A*, representative image of a region of a BPH1 cell expressing GFP-NM2A before photobleaching. The region to be bleached, indicated by the *white circle*, is approximately 1 μm in diameter. The fluorescence recovery within that region over time is shown by the inserts to the RHS, together with the plot of intensity *versus* time, scaled to 1.0 for the plateau. *B*, representation of the t_1/2_ and mobile fraction estimates from multiple cells across at least two independent biological replicates. Individual measurements, superimposed on box and whisker plots are shown. Values were compared by a 1-way ANOVA, in which each of the values was compared to wild type, using a post hoc Dunnett test. Statistically significant differences between mutant constructs and WT are indicated (∗*p* < 0.05; ∗∗∗*p* < 0.001). The *dotted line* depicts the mean value for WT. Averages from three separate experiments. (WT: n = 16, D1424G: n = 11, D1447N n = 11, D1447G, n = 11, D1447H: n = 13, V1516M: n = 9, E1841K: n = 11).

To characterize the turnover of WT and mutant NM2A molecules in filaments in living cells, FRAP was used to analyze filament dynamics using live cells transfected with the GFP-fusion proteins as previously described (24). A small (∼1 μm diameter) area was bleached, and fluorescence recovery was measured (Fig. 6*A*). Analysis of the FRAP data showed that the recovery time was slower (increase in t_1/2_) for all the mutations, although this only reached a statistically significant level for three of the six mutants (Fig. 6*B*). Moreover, the mobile fraction (expressed as %) was decreased in all six mutants, reaching significance for the two mutations in D1424 and for D1447G. These observations suggest that all of the mutations affect filament turnover or stability to some degree. The measured increase in t_1/2_ and decrease in mobile fraction for the mutant sequences may be consistent with a decreased pool of monomers in cells expressing these mutations, as we discuss below.

### Negative-stain EM Counting assay of FL-NM2A shows specific mutations destabilize 10S

**Figure 7:**
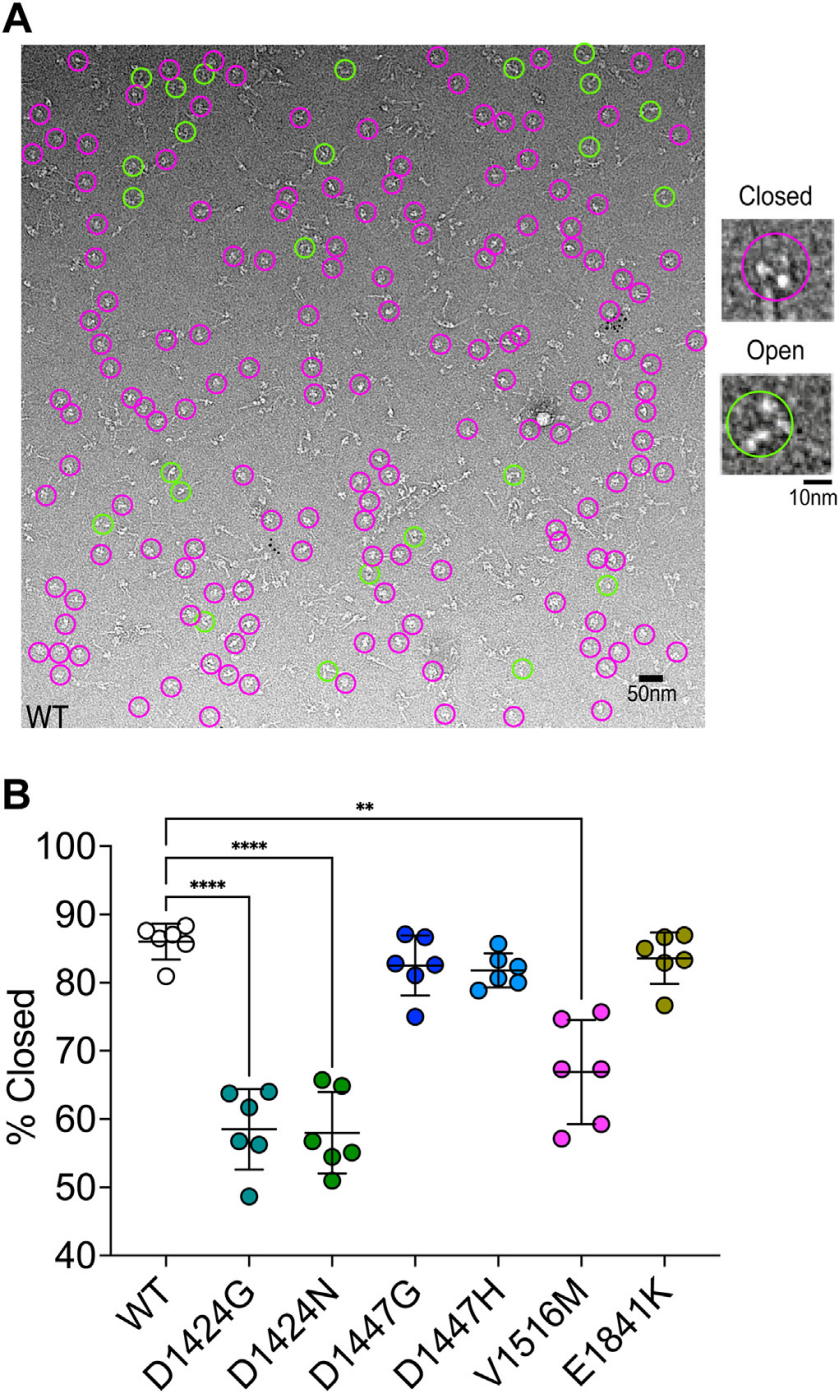
**Analysis of the proportion of full length NM2A molecules that form the shutdown state *in vitro*.***A*, a representative field of view of negatively stained NM2A molecules imaged using electron microscopy. Molecules circled in *magenta* are examples of those that were classified as closed heads, typical of the shutdown state. Molecules circled in *green* are examples of those classified as open heads. Approximately 20% of heads were not able to be classified (see Experimental procedures). *B*, the graph shows the percentage of heads in the closed (shutdown state), determined for two fields of view, and three biological replicates (six fields of view in total) for WT NM2A and each of the mutants. The mean values for each field of view are plotted, together with the mean and SD for the six replicates. Values were compared by a 1-way ANOVA, in which each of the values were compared to wild type, using a post hoc Dunnett test. The total number of molecules analyzed to determine this percentage was 535 (WT), 366 (D1424G), 400 (D1424N), 415 (V1516M), 205 (D1447G), 185 (D1447H) and 194 (E1814K). Mean values were compared using ANOVA and the significance is as shown. ∗∗∗∗*p* < 0.0001; ∗∗*p* < 0.01.

To determine the effect of mutations on the ability of the full-length molecules to form 10S-shutdown molecules, we analyzed negative stain EM images. To avoid disruption of the closed state introduced by the interaction of the molecules with the carbon substrate on the EM grid, molecules were mildly crosslinked (see Experimental procedures). FL molecules were manually classified into one of two subpopulations that were either molecules in the closed (magenta) or open (green) states (Fig. 7*A*). About 20% of the particles in each field of view could not be accurately classified, as a result of stain depth, aggregation of myosin particles, or the unclear shape of the molecule. We then compared the percentage of molecules in the closed (shutdown) state between WT and mutant NM2A molecules (Fig. 7*B*).

We found that, of the classified molecules, WT NM2A mostly adopts the shutdown 10S state in conditions in which we expect this state to form (86.0 ± 2.6%: mean ± SD) (Fig. 7*B*). The percentage of classified molecules that adopt the shutdown state for the mutant isoforms D1424G, D1424N, and V1516M was significantly decreased by ∼20 to 30% compared to WT. The percentage of classified molecules that adopt the shutdown state for the mutant isoforms D1447G and D1447H and E1841K mutants was not significantly different from WT (Fig. 7*B*). This suggests that mutations in D1424 and V1516 decrease the stability of the 10S shutdown state.

## Discussion

In this work, we used a wide range of approaches to understand how specific disease-causing mutations affect the function of NM2A. We selected specific residues (D1424, D1447, and V1516) that we expected might have different effects on the stability of the shutdown state based on their positions in the NM2A homology model, together with a final residue (1841) that does not interact with the blocked head in the homology model but could destabilize the shutdown state through interaction with other regions of the coiled-coil. The model predicted that mutations in D1424 could affect interactions with the blocked head, that mutations in D1447 would not, and that mutations in V1516M might affect the formation and stability of bend-2 which could destabilize the shutdown state. As all the mutations could also affect filament formation, we also analyzed this both *in vitro* and *in vivo*, and we used FRAP to assay filament dynamics. Across all of the assays, each mutation had a greater or lesser effect (summarized in Table 1).

The main effect of the two mutations we tested in D1424 was to destabilize the formation of the shutdown state, with minor effects on secondary structure and filament formation, and little effect on thermal stability (Table 1), generally consistent with an earlier study of D1424N LMM (19). That study showed minor effects on LMM secondary structure and paracrystal formation and no effect on thermal stability. It additionally found that paracrystals formed by D1424N LMM were narrower than WT, similar to our finding that filament widths are narrower. Our homology model predicts that D1424 could form a salt bridge with R702 and/or R705 in the motor domain of the blocked head and that this interaction could contribute to the stability of the shutdown structure (Fig. 2), suggesting why mutations in this residue strongly disrupt the formation of the shutdown state. Interestingly, three different missense mutations have been reported for R702 and one for R705 (Table S1). Both Arg residues are in the SH1 helix which lies between the relay helix and the converter in the motor. The sequence of the SH1 helix in NM2A (VLEGIRICRQ: R702 underlined) is similar to that of the β-cardiac myosin heavy chain (VLEGIRICRK: R703 underlined). A disease-causing mutation in R703 in β-cardiac myosin heavy chain in the equivalent position to R702 in NM2A has also been reported (25). However, β-cardiac myosin does not form a full shutdown state. The coiled-coil tail folds back once at bend-1 to make a minimal interaction with the blocked head motor domain (26). Mutations in R702 are also likely to affect the myosin ATPase in both cardiac and non-muscle myosin (27).

The main effect of the two mutations we tested in D1447 was to increase filament lengths and widths *in vitro*. Mutations did not affect the secondary structure or destabilize the shutdown state. Interestingly, this residue follows on from a patch of positively charged lysine residues (Fig. S1) and leads into a patch of negatively charged glutamate residues that are likely important for regulating packing into filaments (28). As both the mutations tested would decrease the overall charge of this patch, but in particular affect the charge boundary between positive and negative charge, this might help to explain the increased filament widths and lengths we observed. D1447 is in the *e* position of the coiled-coil heptad repeat and could also form a salt bridge with the downstream residue in the *g* position (K1442), five residues away (i, i-5) to help stabilize the coiled-coil. Mutation of D1447 either to Gly (small, uncharged) or His (polar amino acid) could abolish this interaction, if it occurs, and could locally destabilize the coiled-coil. However, D1447H only had a small effect, and D1447G no effect on the secondary structure by CD. Moreover, the coiled-coil is somewhat more open in this region in the 10S structure, and the homology model did not show that this stabilizing interaction does occur, at least in 10S. Moreover, this residue is not predicted to interact with residues in the motor domain of the blocked head in the 10S shutdown state, and as we saw, these two mutations did not have a major effect on 10S stability. Thus, the key effect of mutations in this residue is likely to be on filament packing.

The V1516M mutation destabilized the shutdown 10S state but had little to no effect on the secondary structure or filament packing *in vitro*. The residue V1516 is in a *d* position in the heptad repeat and found close to bend two between segment-1 and segment-2 in the 10S shutdown state. Mutation of the hydrophobic Val residue to the hydrophobic Met residue, introduces a slightly bulkier hydrophobic side chain that could interact with the aromatic residue (His1521) in the downstream *a* position in the heptad (Fig. 2). It is also worth noting that V1516 is followed by one of the three skip residues (G1517), which may contribute to the flexibility of the coiled coil in this region, specifically in the shutdown state (29). Mutation to Met and interaction with His1521 could decrease the flexibility of bend-2, which is important for 10S formation, and in enabling the formation of the latch (interaction of the N-terminal region of RLC with the coiled-coil in the shutdown state) (6). This could help to explain why this mutation predominantly destabilizes the shutdown state.

The E1841K mutation decreased helicity and thermal stability, and increased filament width *in vitro* but did not destabilize the shutdown state. E1841, in an *e* position in the heptad repeat, is found close to the C-terminus of the coiled-coil, which does not directly interact with the motor domain. It could interact with the Gln (Q) residue, five residues downstream (as an i, i-5, *e-g* interaction), which could help stabilize the coiled-coil at this position. This could account for the decreased helicity and thermal stability we saw for LMM constructs with this mutation. A previous study also used CD to show that this mutation destabilized the coiled-coil but did not see a change in thermal stability (19). This may be partly accounted for by the shorter construct (aa1212–1960 of the human NM2A sequence) we used compared to this earlier study (residues 1102–1960) as discussed in the results (19). The small effects we saw for this mutation on filament formation (Table 1) are also broadly consistent with the altered morphology of LMM paracrystals and the increased filament width and length of FL NM2A filaments *in vitro* reported previously (19, 23). This suggests that the change in charge that results from this mutation affects filament packing. The E1841 residue could also be important in 10S formation through its interaction with charged residues (K1129, K1132, K1134, or Arg R1135) in adjacent coiled coils in the 10S molecule. Since current structures for the coiled-coil in the shutdown state have limited resolution in this region, due to the high flexibility of the stacked coiled-coil tail, we cannot yet determine whether this is the case. However, we found that this mutation did not affect 10S stability.

The effects of mutations in NM2A in cells are more complex to interpret. Filament lengths measured from STED images were significantly shorter for mutations in D1424 and V1516M, which both showed an increased t1/2 for fluorescence recovery and decreased mobile fraction, although not always significant. If we assume that the GFP-myosin shutdown state is destabilized in cells, as we observed *in vitro*, this could lead to an increase in GFP-myosin assembled into filaments, and a concomitant decrease in the pool of 10S GFP-myosin. This would be expected to decrease the rate of turnover of myosin in filaments, and thus decrease the rate of fluorescence recovery (increased t1/2). Likewise, a decrease in 10S GFP myosin could lead to shorter filaments if filaments begin to assemble and there are insufficient molecules to generate FL filaments. Alternatively, it is possible that the increased lifetime of D1424 and V1516M mutants, and thus slower turnover of these mutants, as measured by FRAP, shows that these mutants are more likely to be found in the central region of the bipolar filaments, and unlabeled WT myosin is more likely to be found at their ends, leading to the appearance of shorter filaments, as we used the GFP location to measure the ends of the filaments. The mobile fraction for E1814K was also significantly decreased and fluorescence recovery increased, however, filament length was unaltered, suggesting that this mutation mainly affects the dynamics of filament formation rather than the final length of the filaments themselves. The D1447G mutation resulted in slightly longer filaments in cells, reflecting the long filament lengths observed *in vitro*, and the largest reduction in the mobile fraction. Overall, the effects of each mutant on filament length and dynamics could underly the disease phenotype, for example leading to abnormal spreading of activated platelets.

In summary, the segregation of specific myosin mutations into separate classes that exert their pathogenic effects mainly through affecting the stability and formation of the shutdown 10S state, or through effects on filament formation, may be an oversimplification. Mutations in D1424 and V1516 mainly affect the formation of the shutdown state, while the remaining mutations mainly affect filament formation. Nevertheless, filament dynamics in cells are, to some extent, affected by all of the mutations studied. Assuming fluorescence recovery in filaments requires the exchange of monomers into filaments, the results reported here could reflect the effects of mutations on both 10S and filament formation that, in turn, could help to explain the altered dynamics. In diseased cells, where filament dynamics, and specifically the switch from 10S shutdown to active filaments, is important in driving cell shape, the range of effects of the specific mutations we have studied here helps us to understand the observed pathological states.

## Experimental procedures

### Constructs

The LMM constructs contained residues 1212 to 1960 of the human NM2A coding sequence (NCBI: NG_011884.2). LMM was cloned into pGEX-6P-1 (GE Lifesciences), which adds a GST-tag and PreScission protease site at the N-terminus. NM2A full-length heavy chain molecules contained the full-length coding sequence for human NM2A into a pFastBac1 baculovirus polyhedrin promoter vector (ThermoFisher Scientific). An N-terminal FLAG-tag octapeptide (DYKDDDK) was included for the purification of the expressed protein. NM2A molecules were obtained by co-expressing FLAG-tagged MHCs and both light chains; (bovine non-muscle myosin RLC (MYL9) and chicken non-muscle myosin ELC (MYL6)). For expression in mammalian cells, NM2A cDNA was subcloned into a pEGFP-C1 vector (Promega) to introduce an N-terminal GFP tag. All the WT and mutant variant constructs were generated using Genscript.

### Expression/purification

WT/mutant GST-LMM constructs were transformed into *E. coli* Rosetta 2 (Novagen) cells, and a single colony was inoculated into 5 ml of Terrific Broth (TB, Sigma) media to be grown overnight. This overnight culture was added to 1 L of TB, grown at 37 °C until the OD_600_ reached 0.8, induced with the addition of IPTG to a final concentration of 0.5 mM, and expressed at 25 °C overnight. Purification was performed as previously described (Parker *et al.*, J. Mol. Biol. 2018). Briefly, pellets were resuspended in lysis buffer A [PBS, pH 7.5, containing 1 mM DTT, 1 mM EDTA, 200 μg/ml lysozyme, 0.1% Triton X-100, and a tablet of protease cocktail inhibitor tablet (Roche)], sonicated on ice (6 cycles of 10-s on/10-s off). The cell debris was pelleted by centrifugation, with proteins purified by GST-tag affinity chromatography using Glutathione Sepharose 4B (GE Lifesciences). For CD studies, the GST tag was cleaved on-column using PreScission protease. For EM studies, the GST tag was kept to allow filament formation.

Full-length NM2A expression/purification procedures were similar to previous reports (30). Constructs were expressed in Sf9 cells (Invitrogen). pFastBac1 donor plasmids were transformed into DH10-Bac *E. coli* cells, and recombinant bacmid was isolated. Baculovirus was generated by transfecting Sf9 cells with recombinant bacmid DNA and polyethylenimine (Sigma Aldrich) at a ratio of 1:9 in PBS buffer. Cultures were infected with baculovirus at a MOI of 2–5, then were grown for 72 h and the cells were pelleted by centrifugation. Cell pellets were stored at −80 °C.

To purify the protein, frozen pellets were thawed and homogenized on ice using a ground glass homogenizer in buffer B [10 mM MOPS (pH 7.4), 5 mM MgCl_2_, 0.1 mM EGTA) supplemented with 0.5 M NaCl, 2 mM ATP, 0.1 mM phenylmethylsulfonyl fluoride and protease inhibitor cocktail (Roche)] and sonicated on ice (6 cycles of 10-s on/30-s off). Protein was purified using M2 FLAG affinity gel (Sigma) and eluted in buffer B supplemented with 0.5 M NaCl, and 0.5 mg/ml of FLAG peptide (Sigma). The eluted proteins were dialyzed overnight in buffer A supplemented with 0.5 M NaCl and 1 mM DTT. Protein concentration was determined using a NanoDrop spectrophotometer, with the calculated extinction coefficient (0.52 ml mg^−1^ cm^−1^). The protein was flash-frozen in liquid nitrogen in ∼20 μl aliquots and stored in liquid nitrogen until used. Note, NM2A expressed using the baculovirus system is not phosphorylated (22).

### CD

Polymerized LMM was formed as previously described (21). Ultracentrifuged pellets were resuspended in CD buffer (10 mM sodium phosphate, pH 7.4, 500 mM NaCl, and 0.5 mM TCEP). Protein concentration was assessed by Bradford assay (BioRad), and samples were diluted to a final concentration of 0.15 to 0.3 mg/ml.

CD was measured from 260 nm to 190 nm at 1-nm bandwidth, using a 1 mm path length quartz Suprasil cuvette (Hellma) on an APP Chirascan CD spectrometer (Delta Photonics). Low-temperature scans were performed at 10 °C in duplicate for each construct and averaged without smoothing, with at least three experimental runs performed. Buffer background was measured in a sample cuvette and subtracted to generate net spectra.

Thermal denaturation measurements were performed at 1 °C increments from 10 °C to 80 °C at a rate of 0.7 °C/min, with at least three experiments performed per construct. Significant changes in helical content between WT and mutant constructs are indicated by: ∗*p* < 0.05; ∗∗*p* < 0.01; or ∗∗∗*p* < 0.001.

### STED

For STED experiments, BPH1 cells (benign prostate hyperplasia 1) cells (Sigma-Aldrich) were used. This epithelial cell line expresses NM2A, which is nicely ordered into stacks near the cell edges, facilitating imaging. Cultured cells were routinely tested for *mycoplasma* by rt-PCR using a kit (VWR International). Cells were cultured in RPMI supplemented with 10% FCS (fetal calf serum) and penicillin/streptomycin at 37 °C. Cells were transfected with eGFP-NM2A constructs, either WT or mutant. For transfection, cells were trypsinized from flasks and plated onto 13 mm diameter glass coverslips #1.5 (∼170-nm thick). The following day, the cells were transfected using Fugene 6 (Promega), following the manufacturer’s instructions. Briefly, 6 μl of Fugene six was added to ∼92 μl of the medium, vortexed briefly to mix, and incubated for 5 min 2 μg of maxi prepped DNA (ThermoFisher Scientific) was then added, briefly vortexed, and incubated for 20 min. The final volume was 100 μl. 50 μl of transfection mix was used for each coverslip. Cells were incubated for 36 h, and then fixed using 4% paraformaldehyde in PBS for 20 min, washed with PBS, and stored at 4 °C prior to staining.

To stain the cells, they were first permeabilized using 0.5% Triton-X 100 in PBS for 5 to 10 min. Primary antibodies (mouse anti-eGFP (3E6: Thermofisher Scientific) and rabbit anti-NM2A (Biolegend UK: PRB-440P) were diluted at 1/100 in PBS containing 0.2% Triton-X-100. 50 μl of antibody solution was added to each coverslip, and the coverslips incubated for 60 min in a humid chamber. The antibody solution was removed, coverslips were washed in five changes of PBS, and secondary antibodies were added. Secondary antibodies (anti-mouse STAR Red and anti-rabbit STAR Orange (Abberior)) were used at 1/200 dilution in PBS containing 0.2% Triton-X-100. Coverslips were incubated for 60 min, prior to washing in five changes of PBS. Coverslips were mounted in ProLong Gold Antifade (ThermoFisher Scientific). Coverslips were imaged using 2D STED, using a STEDYCON (Abberior), equipped with a x100 objective lens (NA 1.4). The depletion laser was set to provide a nominal resolution of ∼50 nm in both channels. STED images were further deconvolved using Huygens Software (Scientific Volume Imaging).

Filament measurements were carried out using profile plots in ImageJ. Three biological repeats were analyzed using STED, and a minimum of five cells per repeat were imaged, for each of the WT and mutant NM2A-expressing cells. Results from the replicates were combined, graphs generated, and statistical analysis (ANOVA) performed using Prism software (GraphPad).

### FRAP

For FRAP, cells were plated onto glass-bottomed dishes (35 mm glass-bottomed μ-dishes, IBIDI) and transfected as described above. 36 to 48 h later, 1M HEPES solution (ThermoFisher Scientific) was added to the medium to a final concentration of 20 mM, to buffer the medium in air. Cells were imaged on an LSM 880 Airyscan inverted confocal microscope (Zeiss), using the 40x objective lens (NA 1.4) in Airyscan mode. A stage-top incubator maintained the cells at 37 °C. A small circular region (∼1 μm in diameter) was bleached using a 488 nm wavelength laser set to 100% for ∼50 iterations to ensure levels of fluorescence in this region were reduced to very low levels. Fluorescence recovery was then allowed to proceed for approximately 2 min, until recovery had plateaued, at a frame rate of one per second. Data was analyzed using the FRAP plugin in the Zeiss software. Fluorescence recovery curves were fit with a single exponential to obtain values of t_1/2_ and immobile fraction. For each mutation, at least 10 cells over three separate experiments, were analyzed. Results from the replicates were combined, graphs generated, and statistical analysis (ANOVA) was performed using GraphPad Prism.

### EM

GST-LMM and FL NM2A filaments were formed by dilution of samples into low-salt buffer (10 mM phosphate, pH 7.4, 150 mM NaCl, 0.5 mM TCEP), and allowed to form filaments on ice, with a final protein concentration of 200 to 500 nM. 5 μl of sample were applied to a carbon-coated copper grid, produced in-house, which had been glow-discharged for 30 s at 0.38 mBar and 15 mAmp (PELCO easiGlow discharge unit, Ted Pella Inc). The sample was removed and then stained with five drops of 1% uranyl acetate (31). A Tecnai F20 (FEI) TEM microscope was used to image the grids; micrographs were recorded using a 2k × 2k Gatan CCD camera at a pixel size of 0.418 and 0.351 nm for 25 kx and 29 kx, respectively. Image measurements were performed using ImageJ software. For each mutation, grids from at least three separate experiments were analyzed. Statistical analysis (ANOVA) and graph generation was performed using Prism. Significant changes in helical content between WT and mutant peptides are indicated by: ∗*p* < 0.05; ∗∗*p* < 0.01; ∗∗∗*p* < 0.001, or ∗∗∗∗*p* < 0.0001.

10S particles were formed by adding ATP (Sigma-Aldrich) to FL-NM2A, before dilution into a low ionic strength solution. The final conditions were 150 mM KCl, 10 mM MOPS pH 7.2, 0.1 mM EGTA, 2 mM MgCl_2_, and 1 mM ATP. 1 mM Bissulfosuccinimidyl suberate (ThermoFisher) was used to mildly cross-link I μM 10S particles for 30 min at 25 °C and quenched with the addition of pH 8.0 Tris buffer to a final concentration of 100 mM. Protein was diluted to a final concentration of 100 nM. Grids were prepared, imaged, and analyzed as described above.

To estimate the number of molecules in the shutdown state, using the EM data, open and closed particles were determined by identifying particles that had either clear Y-shaped (open) heads or compact heads after following the path of the tails. This stringent approach was taken to avoid erroneous identification of shutdown or open heads. Approximately 20% of particles per micrograph could not be unambiguously identified based on these criteria as a result of variations in stain depth, aggregation of molecules, or molecules with similar conformations lying at oblique angles. This analysis was performed manually using printed copies (A3) of a minimum of two fields of view, for each of three separate biological replicates for the WT and each mutant construct (6 in total per construct). It was performed by one person, using a blinded approach (printouts were labeled with a reference number that did not identify which construct had been imaged). The percentage of the total number of classified heads that had adopted the shutdown state (compact heads) was calculated and compared between WT and each of the mutants. Statistical significance was determined using a one-way ANOVA with a post hoc Dunnett’s test in GraphPad Prizm. N (total number of molecules analyzed) was 535 (WT), 366 (D1424G), 400 (D1424N), 415 (V1516M), 205 (D1447G), 185 (D1447H), and 194 (E1814K).

### Homology model

To create the homology model of NM2A from our previously published SMM structure, residues were replaced in Coot and molecular dynamics simulations were performed using the GBSA implicit solvent model in conjunction with the Amber FF14SB force field as described previously (6). Briefly, hydrogen atoms were automatically added to our refined coot model with the Leap module in the AMBER package (32). The system was then minimized using a steepest descent minimization followed by a conjugate gradient algorithm and then thermalized, where the temperature is increased in a stepwise manner with the constraining forces slowly moved over the number of steps. Shake (33) found within the AMBER package, which constrains bond length, was used to constrain all bonds involving hydrogen atoms with a time step of 2 fs and a 10.0 Å cut-off for non-bonded interactions. The simulation was run for 2 ns using backbone restraints 10 kcal/mol∗Ang^−2^ to allow the formation of ionic interactions without compromising the peptide backbone structure. The final frame of the simulation was analyzed and used as the basis for our pseudo-atomic NM2A model.

## Data availability

All the data are contained within the manuscript.

## Supporting information

This article contains supporting information.

## Conflict of interest

The authors declare that they have no conflicts of interest with the contents of this article.

## References

[1] Conti M.A., Adelstein R.S. (2008). Nonmuscle myosin II moves in new directions. J. Cell Sci..

[2] Craig R., Smith R., Kendrick-Jones J. (1983). Light-chain phosphorylation controls the conformation of vertebrate non-muscle and smooth muscle myosin molecules. Nature.

[3] Cross R.A., Jackson A.P., Citi S., Kendrick-Jones J., Bagshaw C.R. (1988). Active site trapping of nucleotide by smooth and non-muscle myosins. J. Mol. Biol..

[4] Moussavi R.S., Kelley C.A., Adelstein R.S. (1993). Phosphorylation of vertebrate nonmuscle and smooth muscle myosin heavy chains and light chains. Mol. Cell. Biochem..

[5] Heissler S.M., Arora A.S., Billington N., Sellers J.R., Chinthalapudi K. (2021). Cryo-EM structure of the autoinhibited state of myosin-2. Sci. Adv..

[6] Scarff C.A., Carrington G., Casas-Mao D., Chalovich J.M., Knight P.J., Ranson N.A. (2020). Structure of the shutdown state of myosin-2. Nature.

[7] Yang S., Tiwari P., Lee K.H., Sato O., Ikebe M., Padrón R. (2020). Cryo-EM structure of the inhibited (10S) form of myosin II. Nature.

[8] Lupas A. (1996). Coiled coils: new structures and new functions. Trends Biochem. Sci..

[9] Straussman R., Squire J.M., Ben-Ya'acov A., Ravid S. (2005). Skip residues and charge interactions in myosin II coiled-coils: implications for molecular packing. J. Mol. Biol..

[10] Ikebe M., Komatsu S., Woodhead J.L., Mabuchi K., Ikebe R., Saito J. (2001). The tip of the coiled-coil rod determines the filament formation of smooth muscle and nonmuscle myosin. J. Biol. Chem..

[11] Pecci A., Ma X., Savoia A., Adelstein R.S. (2018). MYH9: structure, functions and role of non-muscle myosin IIA in human disease. Gene.

[12] Bury L., Megy K., Stephens J.C., Grassi L., Greene D., Gleadall N. (2020). Next-generation sequencing for the diagnosis of MYH9-RD: predicting pathogenic variants. Hum. Mutat..

[13] Pecci A., Klersy C., Gresele P., Lee K.J., De Rocco D., Bozzi V. (2014). MYH9-related disease: a novel prognostic model to predict the clinical evolution of the disease based on genotype-phenotype correlations. Hum. Mutat..

[14] Murakami N., Elzinga M. (1992). Immunohistochemical studies on the distribution of cellular myosin II isoforms in brain and aorta. Cell Motil. Cytoskeleton.

[15] Daniel J.L., Molish I.R., Rigmaiden M., Stewart G. (1984). Evidence for a role of myosin phosphorylation in the initiation of the platelet shape change response. J. Biol. Chem..

[16] Zelena A., Blumberg J., Probst D., Gerasimaite R., Lukinavicius G., Schwarz U.S. (2023). Force generation in human blood platelets by filamentous actomyosin structures. Biophys. J..

[17] Asensio-Juarez G., Llorente-Gonzalez C., Vicente-Manzanares M. (2020). Linking the landscape of MYH9-related diseases to the molecular mechanisms that control non-muscle myosin II-A function in Cells. Cells.

[18] Smith A.S., Pal K., Nowak R.B., Demenko A., Zaninetti C., Da Costa L. (2019). MYH9-related disease mutations cause abnormal red blood cell morphology through increased myosin-actin binding at the membrane. Am. J. Hematol..

[19] Franke J.D., Dong F., Rickoll W.L., Kelley M.J., Kiehart D.P. (2005). Rod mutations associated with MYH9-related disorders disrupt nonmuscle myosin-IIA assembly. Blood.

[20] Wolny M., Colegrave M., Colman L., White E., Knight P.J., Peckham M. (2013). Cardiomyopathy mutations in the tail of beta-cardiac myosin modify the coiled-coil structure and affect integration into thick filaments in muscle sarcomeres in adult cardiomyocytes. J. Biol. Chem..

[21] Parker F., Batchelor M., Wolny M., Hughes R., Knight P.J., Peckham M. (2018). A1603P and K1617del, mutations in beta-cardiac myosin heavy chain that cause laing early-onset distal myopathy, affect secondary structure and filament formation *In Vitro* and *In Vivo*. J. Mol. Biol..

[22] Billington N., Wang A., Mao J., Adelstein R.S., Sellers J.R. (2013). Characterization of three full-length human nonmuscle myosin II paralogs. J. Biol. Chem..

[23] Pal K., Nowak R., Billington N., Liu R., Ghosh A., Sellers J.R. (2020). Megakaryocyte migration defects due to nonmuscle myosin IIA mutations underlie thrombocytopenia in MYH9-related disease. Blood.

[24] Lopata A., Hughes R., Tiede C., Heissler S.M., Sellers J.R., Knight P.J. (2018). Affimer proteins for F-actin: novel affinity reagents that label F-actin in live and fixed cells. Sci. Rep..

[25] Colegrave M., Peckham M. (2014). Structural implications of beta-cardiac myosin heavy chain mutations in human disease. Anat. Rec. (Hoboken).

[26] Jung H.S., Komatsu S., Ikebe M., Craig R. (2008). Head-head and head-tail interaction: a general mechanism for switching off myosin II activity in cells. Mol. Biol. Cell.

[27] Bobkova E.A., Bobkov A.A., Levitsky D.I., Reisler E. (1999). Effects of SH1 and SH2 modifications on myosin: similarities and differences. Biophys. J..

[28] Ricketson D., Johnston C.A., Prehoda K.E. (2010). Multiple tail domain interactions stabilize nonmuscle myosin II bipolar filaments. Proc. Natl. Acad. Sci. U. S. A..

[29] Taylor K.C., Buvoli M., Korkmaz E.N., Buvoli A., Zheng Y., Heinze N.T. (2015). Skip residues modulate the structural properties of the myosin rod and guide thick filament assembly. Proc. Natl. Acad. Sci. U. S. A..

[30] Melli L., Billington N., Sun S.A., Bird J.E., Nagy A., Friedman T.B. (2018). Bipolar filaments of human nonmuscle myosin 2-A and 2-B have distinct motile and mechanical properties. Elife.

[31] Scarff C.A., Fuller M.J.G., Thompson R.F., Iadanza M.G. (2018). Variations on negative stain electron microscopy methods: tools for tackling challenging systems. J. Vis. Exp..

[32] Case D.A., Belfon K., Ben-Shalom I.Y., Brozell S.R., Cerutti D.S., Cheatham I. (2020).

[33] Ryckaert J.-P., Ciccotti G., Berendsen H.J.C. (1977). Numerical integration of the cartesian equations of motion of a system with constraints: molecular dynamics of n-alkanes. J. Comput. Phys..

