# Supplementary material for: Effects of specific disease mutations in non-muscle myosin 2A on its structure and function
Source: J Biol Chem. 2023 Nov 30;300(1):105514. doi: 10.1016/j.jbc.2023.105514 (PMC10770755; doi:10.1016/j.jbc.2023.105514)
Supplement: Supporting Figure S1 and Table S1 [file mmc1.docx]

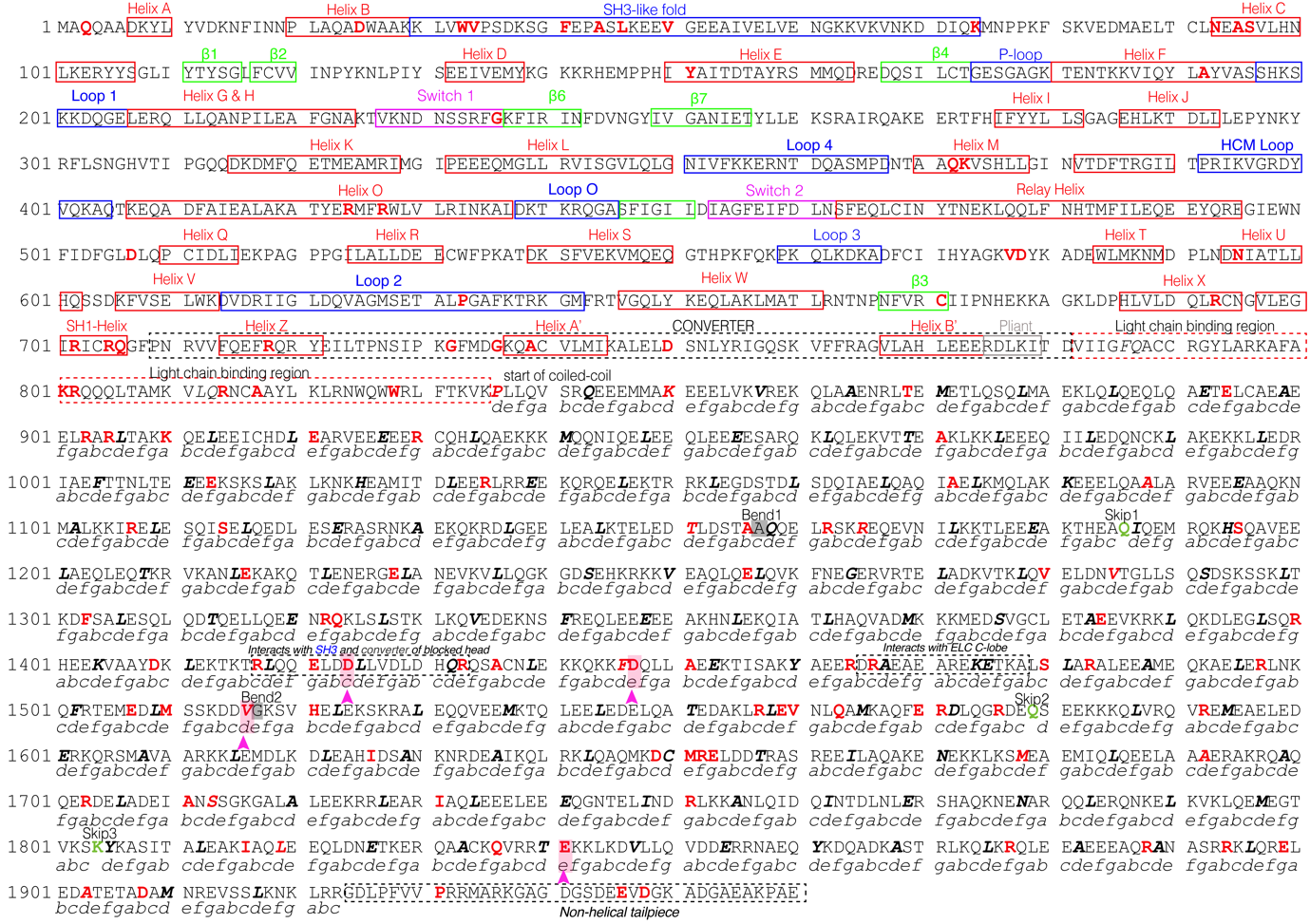


**Supplemental Figure 1**: Mutations in NM2A. Mutations (red, bold, see supplementary Table 1) plotted onto the linear sequence. Domains within the motor domain are as indicated. The start of the coiled coil is indicated, with the heptad sequence below, and the positions of the three skip residues as indicated. The *d* positions in the heptad motif are indicated in bold and italic font. Mutations in these regions are coloured red, bold and italic. The positions of the 4 mutated residues investigated here (1424, 1447, 1516 and 1841) are indicated by a pink box.

**Supplemental Table 1:** Tabulation of the mutations plotted in Figure 1 together with the disease reported for each mutation (all in the umbrella term of MYH9-related disease) with the citation for each reported mutation.

| Mutation | Region | Disease | Reference |
| --- | --- | --- | --- |
| Q3E | N-terminal | B-cell lymphoid neoplasm | (1) |
| D25N | SH3-like fold | Neurodevelopmental disorder | (2) |
| W33R | SH3-like fold | May-Hegglin anomaly | (3, 4) |
| W33C | SH3-like fold | MYH9 related disease | (5) |
| W33G | SH3-like fold | MYH9 related disease | (6) |
| V34E | SH3-like fold | MYH9 related disease | (7) |
| V34G | SH3-like fold | MYH9 related disease | (8) |
| F41I | SH3-like fold | MYH9 related disease | (9) |
| F41L | SH3-like fold | MYH9 related disease | (9) |
| F41S | SH3-like fold | MYH9 related disease | (6) |
| A44P | SH3-like fold | MYH9 related disease | (7) |
| L46F | SH3-like fold | MYH9 related disease | (10) |
| E52K | SH3-like fold | MYH9 related disease | (11) |
| V56M | SH3-like fold | MYH9 related disease | (2) |
| K74E | SH3-like fold | MYH9 related disease | (12) |
| N93D | Motor | Macrothrombocytopaenia with leukocyte inclusions | (13) |
| N93K | Motor | May-Hegglin anomaly | (14) |
| A95D | Motor | Macrothrombocytopaenia with leukocyte inclusions | (13) |
| A95T | Motor | Macrothrombocytopaenia with leukocyte inclusions | (15) |
| A95V | Motor | MYH9 related disease | (7) |
| S96L | Motor | Epstein/Fechtner Syndrome | (16) |
| Y151C | Motor | Non-syndromic cleft lip/palate | (17) |
| A192V | Motor | Neurodevelopmental disorder | (2) |
| G236C | Motor | MYH9-associated elastin aggregation | (18) |
| R272Q | Motor | Bipolar disorder | (19) |
| G312R | Motor | Neurodevelopmental disorder | (2) |
| D315Y | Motor | Neurodevelopmental disorder | (2) |
| G350S | Motor | Neurodevelopmental disorder | (2) |
| A363V | Motor | Neurodevelopmental disorder | (2) |
| Q372R | Motor | MYH9 related disease | (7) |
| K373N | Motor | May-Hegglin anomaly/Sebastian Syndrome | (20) |
| R424Q | Motor | MYH9 related disease | (21) |
| R427C | Motor | Neurodevelopmental disorder | (2) |
| D507N | Motor | Ovarian cancer? | (22) |
| V577A | Motor | Kidney Disease | (23) |
| D578Y | Motor | MYH9-related disease | (24) |
| N595S | Motor | Nephrotic Syndrome | (25) |
| P633S | Motor | Deafness | (26) |
| C671Y | Motor | MYH9-associated elastin aggregation | (18) |
| R693C | Motor | Neurodevelopmental disorder | (2) |
| R702C | Motor | Fechtner Syndrome | (14) |
| R702S | Motor | Alport syndrome with macrothrombocytopaenia | (20) |
| R702H | Motor | MYH9-related disease | (8) |
| R705H | Motor | Deafness, autosomal dominant 17 | (27) |
| Q706E | Motor | May-Hegglin anomaly | (28) |
| R718W | Motor | MYH9-related disease | (29) |
| G732S | Motor | Neurodevelopmental disorder | (2) |
| G736R | Motor | Neurodevelopmental disorder | (2) |
| A739V | Motor | Neurodevelopmental disorder | (2) |
| D750N | Motor | Neurodevelopmental disorder | (2) |
| K801N | Motor | Platelet count disorder | (11) |
| R802Q | Motor | Hearing loss, non-syndromic | (30) |
| R814Q | Motor | Nephrotic syndrome, | (31) |
| A817S | Motor | MYH9-related disease | (32) |
| W828R | Motor | MYH9-related disease | (33) |
| P836L | Start of CC (*d*) | Deafness | (34) |
| P836Q | Start of CC (*d*) | Neurodevelopmental disorder | (2) |
| K850E | CC | MYH9-related disease | (7) |
| T869M | CC | Non-syndromic cleft lip/palate | (17) |
| E894K | CC | MYH9-related disease | (7) |
| R903G | CC | MYH9-related disease | (35) |
| R905H | CC | Autism spectrum disorder | (36) |
| K910Q | CC | Fechtner Syndrome | (37) |
| E921K | CC | MYH9-related disease | (6) |
| R930H | CC | Hearing Loss | (38) |
| A971V | CC | MYH9-related disease | (24) |
| E1013A | CC | Neurodevelopmental disorder | (2) |
| R1035C | CC | Neurodevelopmental disorder | (2) |
| A1072V | CC | Nephrotic syndrome, | (25) |
| A1088T | CC | Non-syndromic cleft lip/palate | (17) |
| R1107Q | CC | Non-syndromic cleft lip/palate | (17) |
| S1114P | CC | Alport syndrome with macrothrombocytopaenia | (20) |
| T1151M | CC | May-Hegglin anomaly | (32) |
| T1155A | CC | MYH9-related disease | (29) |
| T1155I | CC | May-Hegglin anomaly | (39) |
| R1162S | CC | MYH9-related disease | (40) |
| R1162T | CC | MYH9-related disease | (41) |
| R1165C | CC | Sebastian Syndrome | (14) |
| R1165L | CC | Macrothrombocytopaenia with leukocyte inclusions | (15) |
| S1195L | CC | MYH9-related disease | (6) |
| E1216K | CC | Neurodevelopmental disorder | (2) |
| E1228K | CC | Hearing Loss | (42) |
| E1251K | CC | MYH9-related disease | (7) |
| E1256K | CC | Deafness | (43) |
| V1280M | CC | Nephrotic Syndrome | (25) |
| V1285M | CC | Schizophrenia | (44) |
| F1303L | CC | Hearing Loss | (45) |
| R1322Q | CC | Neurodevelopmental disorder | (2) |
| R1322W | CC | Neurodevelopmental disorder | (2) |
| Q1323H | CC | Hearing Loss | (46) |
| E1384Q | CC | Congenital cataract, autosomal | (47) |
| R1400W | CC | Epstein Syndrome | (16) |
| D1409N | CC | MYH9-related disease | (24) |
| R1417Q | CC (BH interact) | MYH9-related disease | (35) |
| E1421A | CC (BH interact) | MYH9-related disease | (6) |
| E1421K | CC (BH interact) | MYH9-related disease | (40) |
| D1424N | CC (BH interact) | May-Hegglin Anomaly | (15) |
| D1424E | CC (BH interact) | MYH9-related disease | (7) |
| D1424G | CC (BH interact) | MYH9-related disease | (7) |
| D1424H | CC (BH interact) | Fechtner Syndrome | (14) |
| D1424Y | CC (BH interact) | Macrothrombocytopaenia with leucocyte inclusions | (15) |
| R1433C | CC (BH interact) | Hearing Loss | (48) |
| R1433H | CC (BH interact) | Neurodevelopmental disorder | (2) |
| A1436T | CC | MYH9-related disease | (7) |
| F1446L | CC (*d*) | MYH9-related disease | (49) |
| D1447G | CC (*e*) | May-Hegglin Anomaly | (50) |
| D1447H | CC (*e*) | May-Hegglin Anomaly | (50) |
| D1447Y | CC (*e*) | MYH9-related disease | (8) |
| D1447V | CC (*e*) | MYH9-related disease | (51) |
| A1451V | CC | Hearing Loss | (52) |
| R1464H | CC | Nephrotic syndrome | (53) |
| R1466W | CC | B-cell lymphoid neoplasm | (1) |
| E1475K | CC | MYH9-related disease | (7) |
| S1480W | CC | MYH9-related disease | (24) |
| R1483Q | CC | Neurodevelopmental disorder | (2) |
| R1497W | CC | Hearing Loss | (54) |
| E1507Q | CC | Platelet Count Disorder | (11) |
| M1510R | CC | Neurodevelopmental disorder | (11) |
| V1516L | CC *(d)* | May-Hegglin Anomaly | (55) |
| V1516M | CC *(d)* | MYH9-related disease | (56) |
| H1521R | CC | Thrombocytopaenia | (57) |
| R1557L | CC | MYH9-related disease | (56) |
| E1559G | CC | Hearing Loss | (38) |
| V1560G | CC | MYH9-related disease | (7) |
| Q1563K | CC | MYH9-related disease | (7) |
| E1570K | CC | Neurodevelopmental disorder | (2) |
| R1571Q | CC | Autism | (58) |
| R1576W | CC | Neurodevelopmental disorder | (2) |
| R1592Q | CC | Neurodevelopmental disorder | (2) |
| I1626V | CC | Nonsyndromic orofacial clefts | (59) |
| D1649G | CC | MYH9-related disease | (6) |
| M1651T | CC | MYH9-related disease | (60) |
| R1652H | CC | Neurodevelopmental disorder | (2) |
| E1653G | CC | Bladder exstrophy | (61) |
| M1678V | CC | Neurodevelopmental disorder | (6) |
| A1692T | CC | Platelet count disorder | (11) |
| R1703P | CC | B-cell lymphoid neoplasm | (1) |
| A1711T | CC | Predominantly antibody deficiency | (62) |
| S1713G | CC | MYH9-related disease | (63) |
| R1730H | CC | Acute lymphoblastic leukaemia | (64) |
| R1730C | CC | Hearing Loss | (45) |
| D1750N | CC | Neurodevelopmental disorder | (2) |
| I1816V | CC | Alport syndrome with macrothrombocytopaneia | (15) |
| L1819R | CC | Hearing loss, age related | (65) |
| Q1836R | CC | MYH9-related disease | (66) |
| E1841K | CC | May-Hegglin Anomaly | (14) |
| R1877Q | CC | MYH9-related disease | (7) |
| R1877W | CC | Neurodevelopmental disorder | (2) |
| R1888W | CC | Neurodevelopmental disorder | (2) |
| R1894W | CC | Neurodevelopmental disorder | (2) |
| E1899K | CC | Neurodevelopmental disorder | (2) |
| A1903T | CC | Intellectual disability | (36) |
| D1908N | CC | Non-Syndromic cleft lip/palate | (17) |
| V1930M | Non-helical tail piece | Modified of dilated cardiomyopathy | (67) |
| E1946K | Non-helical tail piece | Macrothrombocytopaenia | (68) |
| D1948N | Non-helical tail piece | Macrothrombocytopaenia | (68) |

Supplementary Table 1: Mutations for MYH9 (Source HGMD: Human Genome Mutation Database: Dec. 2022). All of the disease fall into the general category of MYH(-related disease. The description left in refers to how they were first described in the literature. Mutations cover bleeding disorders, hearing loss, kidney and neurodevelopmental disorders.

1. Mosquera Orgueira, A., Cid Lopez, M., Peleteiro Raindo, A., Diaz Arias, J. A., Antelo Rodriguez, B., Bao Perez, L. *et al.* (2021) Detection of Rare Germline Variants in the Genomes of Patients with B-Cell Neoplasms Cancers (Basel) **13**, 10.3390/cancers13061340

2. Wang, T., Hoekzema, K., Vecchio, D., Wu, H., Sulovari, A., Coe, B. P. *et al.* (2020) Large-scale targeted sequencing identifies risk genes for neurodevelopmental disorders Nat Commun **11**, 4932 10.1038/s41467-020-18723-y

3. Jang, M. J., Park, H. J., Chong, S. Y., Huh, J. Y., Kim, I. H., Jang, J. H. *et al.* (2012) A Trp33Arg mutation at exon 1 of the MYH9 gene in a Korean patient with May-Hegglin anomaly Yonsei Med J **53**, 662-666 10.3349/ymj.2012.53.3.662

4. Sun, X. H., Wang, Z. Y., Cao, L. J., Su, J., Jiang, M. H., Wang, G. F. *et al.* (2012) [Clinical features and gene analyses of six patients with MYH9-related disease] Zhonghua Xue Ye Xue Za Zhi **33**, 552-555, <https://www.ncbi.nlm.nih.gov/pubmed/22967416>

5. Kahr, W. H., Savoia, A., Pluthero, F. G., Li, L., Christensen, H., De Rocco, D. *et al.* (2009) Megakaryocyte and platelet abnormalities in a patient with a W33C mutation in the conserved SH3-like domain of myosin heavy chain IIA Thromb Haemost **102**, 1241-1250 10.1160/TH09-02-0119

6. Bury, L., Megy, K., Stephens, J. C., Grassi, L., Greene, D., Gleadall, N. *et al.* (2020) Next-generation sequencing for the diagnosis of MYH9-RD: Predicting pathogenic variants Hum Mutat **41**, 277-290 10.1002/humu.23927

7. Saposnik, B., Binard, S., Fenneteau, O., Nurden, A., Nurden, P., Hurtaud-Roux, M. F. *et al.* (2014) Mutation spectrum and genotype-phenotype correlations in a large French cohort of MYH9-Related Disorders Mol Genet Genomic Med **2**, 297-312 10.1002/mgg3.68

8. De Rocco, D., Zieger, B., Platokouki, H., Heller, P. G., Pastore, A., Bottega, R. *et al.* (2013) MYH9-related disease: five novel mutations expanding the spectrum of causative mutations and confirming genotype/phenotype correlations Eur J Med Genet **56**, 7-12 10.1016/j.ejmg.2012.10.009

9. Smith, A. S., Pal, K., Nowak, R. B., Demenko, A., Zaninetti, C., Da Costa, L. *et al.* (2019) MYH9-related disease mutations cause abnormal red blood cell morphology through increased myosin-actin binding at the membrane Am J Hematol **94**, 667-677 10.1002/ajh.25472

10. Chatterjee, R., Hoffman, M., Cliften, P., Seshan, S., Liapis, H., andJain, S. (2013) Targeted exome sequencing integrated with clinicopathological information reveals novel and rare mutations in atypical, suspected and unknown cases of Alport syndrome or proteinuria PLoS One **8**, e76360 10.1371/journal.pone.0076360

11. Downes, K., Megy, K., Duarte, D., Vries, M., Gebhart, J., Hofer, S. *et al.* (2019) Diagnostic high-throughput sequencing of 2396 patients with bleeding, thrombotic, and platelet disorders Blood **134**, 2082-2091 10.1182/blood.2018891192

12. Kanematsu, T., Suzuki, N., Yoshida, T., Kishimoto, M., Aoki, T., Ogawa, M. *et al.* (2016) A case of MYH9 disorders caused by a novel mutation (p.K74E) Ann Hematol **95**, 161-163 10.1007/s00277-015-2506-9

13. de Rocco, D., Heller, P. G., Girotto, G., Pastore, A., Glembotsky, A. C., Marta, R. F. *et al.* (2009) MYH9 related disease: a novel missense Ala95Asp mutation of the MYH9 gene Platelets **20**, 598-602 10.3109/09537100903349620

14. Seri, M., Cusano, R., Gangarossa, S., Caridi, G., Bordo, D., Lo Nigro, C. *et al.* (2000) Mutations in MYH9 result in the May-Hegglin anomaly, and Fechtner and Sebastian syndromes. The May-Heggllin/Fechtner Syndrome Consortium Nat Genet **26**, 103-105 10.1038/79063

15. Kunishima, S., Matsushita, T., Kojima, T., Amemiya, N., Choi, Y. M., Hosaka, N. *et al.* (2001) Identification of six novel MYH9 mutations and genotype-phenotype relationships in autosomal dominant macrothrombocytopenia with leukocyte inclusions J Hum Genet **46**, 722-729 10.1007/s100380170007

16. Arrondel, C., Vodovar, N., Knebelmann, B., Grunfeld, J. P., Gubler, M. C., Antignac, C. *et al.* (2002) Expression of the nonmuscle myosin heavy chain IIA in the human kidney and screening for MYH9 mutations in Epstein and Fechtner syndromes J Am Soc Nephrol **13**, 65-74 10.1681/ASN.V13165

17. Peng, H. H., Chang, N. C., Chen, K. T., Lu, J. J., Chang, P. Y., Chang, S. C. *et al.* (2016) Nonsynonymous variants in MYH9 and ABCA4 are the most frequent risk loci associated with nonsyndromic orofacial cleft in Taiwanese population BMC Med Genet **17**, 59 10.1186/s12881-016-0322-2

18. Fewings, E., Ziemer, M., Hortnagel, K., Reicherter, K., Larionov, A., Redman, J. *et al.* (2019) Malta (MYH9 Associated Elastin Aggregation) Syndrome: Germline Variants in MYH9 Cause Rare Sweat Duct Proliferations and Irregular Elastin Aggregations J Invest Dermatol **139**, 2238-2241 e2236 10.1016/j.jid.2019.03.1151

19. Nishioka, M., Kazuno, A. A., Nakamura, T., Sakai, N., Hayama, T., Fujii, K. *et al.* (2021) Systematic analysis of exonic germline and postzygotic de novo mutations in bipolar disorder Nat Commun **12**, 3750 10.1038/s41467-021-23453-w

20. Heath, K. E., Campos-Barros, A., Toren, A., Rozenfeld-Granot, G., Carlsson, L. E., Savige, J. *et al.* (2001) Nonmuscle myosin heavy chain IIA mutations define a spectrum of autosomal dominant macrothrombocytopenias: May-Hegglin anomaly and Fechtner, Sebastian, Epstein, and Alport-like syndromes Am J Hum Genet **69**, 1033-1045 10.1086/324267

21. Groopman, E. E., Marasa, M., Cameron-Christie, S., Petrovski, S., Aggarwal, V. S., Milo-Rasouly, H. *et al.* (2019) Diagnostic Utility of Exome Sequencing for Kidney Disease N Engl J Med **380**, 142-151 10.1056/NEJMoa1806891

22. Kanchi, K. L., Johnson, K. J., Lu, C., McLellan, M. D., Leiserson, M. D., Wendl, M. C. *et al.* (2014) Integrated analysis of germline and somatic variants in ovarian cancer Nat Commun **5**, 3156 10.1038/ncomms4156

23. Schrezenmeier, E., Kremerskothen, E., Halleck, F., Staeck, O., Liefeldt, L., Choi, M. *et al.* (2021) The underestimated burden of monogenic kidney disease in adults waitlisted for kidney transplantation Genet Med **23**, 1219-1224 10.1038/s41436-021-01127-8

24. Westbury, S. K., Turro, E., Greene, D., Lentaigne, C., Kelly, A. M., Bariana, T. K. *et al.* (2015) Human phenotype ontology annotation and cluster analysis to unravel genetic defects in 707 cases with unexplained bleeding and platelet disorders Genome Med **7**, 36 10.1186/s13073-015-0151-5

25. Sen, E. S., Dean, P., Yarram-Smith, L., Bierzynska, A., Woodward, G., Buxton, C. *et al.* (2017) Clinical genetic testing using a custom-designed steroid-resistant nephrotic syndrome gene panel: analysis and recommendations J Med Genet **54**, 795-804 10.1136/jmedgenet-2017-104811

26. Li, Y., Su, J., Zhang, J., Pei, J., Li, D., Zhang, Y. *et al.* (2021) Targeted next-generation sequencing of deaf patients from Southwestern China Mol Genet Genomic Med **9**, e1660 10.1002/mgg3.1660

27. Lalwani, A. K., Goldstein, J. A., Kelley, M. J., Luxford, W., Castelein, C. M., andMhatre, A. N. (2000) Human nonsyndromic hereditary deafness DFNA17 is due to a mutation in nonmuscle myosin MYH9 Am J Hum Genet **67**, 1121-1128 10.1016/S0002-9297(07)62942-5

28. Otsubo, K., Kanegane, H., Nomura, K., Ogawa, J., Miyawaki, T., andKunishima, S. (2006) Identification of a novel MYH9 mutation in a patient with May-Hegglin anomaly Pediatr Blood Cancer **47**, 968-969 10.1002/pbc.20879

29. Pecci, A., Panza, E., Pujol-Moix, N., Klersy, C., Di Bari, F., Bozzi, V. *et al.* (2008) Position of nonmuscle myosin heavy chain IIA (NMMHC-IIA) mutations predicts the natural history of MYH9-related disease Hum Mutat **29**, 409-417 10.1002/humu.20661

30. Sommen, M., Schrauwen, I., Vandeweyer, G., Boeckx, N., Corneveaux, J. J., van den Ende, J. *et al.* (2016) DNA Diagnostics of Hereditary Hearing Loss: A Targeted Resequencing Approach Combined with a Mutation Classification System Hum Mutat **37**, 812-819 10.1002/humu.22999

31. Nagano, C., Yamamura, T., Horinouchi, T., Aoto, Y., Ishiko, S., Sakakibara, N. *et al.* (2020) Comprehensive genetic diagnosis of Japanese patients with severe proteinuria Sci Rep **10**, 270 10.1038/s41598-019-57149-5

32. Yang, L., Liu, X., Li, Z., Zhang, P., Wu, B., Wang, H. *et al.* (2020) Genetic aetiology of early infant deaths in a neonatal intensive care unit J Med Genet **57**, 169-177 10.1136/jmedgenet-2019-106221

33. Rabbolini, D. J., Chun, Y., Latimer, M., Kunishima, S., Fixter, K., Valecha, B. *et al.* (2018) Diagnosis and treatment of MYH9-RD in an Australasian cohort with thrombocytopenia Platelets **29**, 793-800 10.1080/09537104.2017.1356920

34. Neveling, K., Feenstra, I., Gilissen, C., Hoefsloot, L. H., Kamsteeg, E. J., Mensenkamp, A. R. *et al.* (2013) A post-hoc comparison of the utility of sanger sequencing and exome sequencing for the diagnosis of heterogeneous diseases Hum Mutat **34**, 1721-1726 10.1002/humu.22450

35. Bastida, J. M., Lozano, M. L., Benito, R., Janusz, K., Palma-Barqueros, V., Del Rey, M. *et al.* (2018) Introducing high-throughput sequencing into mainstream genetic diagnosis practice in inherited platelet disorders Haematologica **103**, 148-162 10.3324/haematol.2017.171132

36. Li, J., Wang, L., Guo, H., Shi, L., Zhang, K., Tang, M. *et al.* (2017) Targeted sequencing and functional analysis reveal brain-size-related genes and their networks in autism spectrum disorders Mol Psychiatry **22**, 1282-1290 10.1038/mp.2017.140

37. Seri, M., Pecci, A., Di Bari, F., Cusano, R., Savino, M., Panza, E. *et al.* (2003) MYH9-related disease: May-Hegglin anomaly, Sebastian syndrome, Fechtner syndrome, and Epstein syndrome are not distinct entities but represent a variable expression of a single illness Medicine (Baltimore) **82**, 203-215 10.1097/01.md.0000076006.64510.5c

38. Chen, S., Dong, C., Wang, Q., Zhong, Z., Qi, Y., Ke, X. *et al.* (2016) Targeted Next-Generation Sequencing Successfully Detects Causative Genes in Chinese Patients with Hereditary Hearing Loss Genet Test Mol Biomarkers **20**, 660-665 10.1089/gtmb.2016.0051

39. Kelley, M. J., Jawien, W., Ortel, T. L., andKorczak, J. F. (2000) Mutation of MYH9, encoding non-muscle myosin heavy chain A, in May-Hegglin anomaly Nat Genet **26**, 106-108 10.1038/79069

40. Zaninetti, C., De Rocco, D., Giangregorio, T., Bozzi, V., Demeter, J., Leoni, P. *et al.* (2019) MYH9-Related Thrombocytopenia: Four Novel Variants Affecting the Tail Domain of the Non-Muscle Myosin Heavy Chain IIA Associated with a Mild Clinical Evolution of the Disorder Hamostaseologie **39**, 87-94 10.1055/s-0038-1645840

41. Vettore, S., De Rocco, D., Gerber, B., Scandellari, R., Bianco, A. M., Balduini, C. L. *et al.* (2010) A G to C transversion at the last nucleotide of exon 25 of the MYH9 gene results in a missense mutation rather than in a splicing defect Eur J Med Genet **53**, 256-260 10.1016/j.ejmg.2010.06.010

42. Schrauwen, I., Melegh, B. I., Chakchouk, I., Acharya, A., Nasir, A., Poston, A. *et al.* (2019) Hearing impairment locus heterogeneity and identification of PLS1 as a new autosomal dominant gene in Hungarian Roma Eur J Hum Genet **27**, 869-878 10.1038/s41431-019-0372-y

43. Wu, C. C., Lin, Y. H., Lu, Y. C., Chen, P. J., Yang, W. S., Hsu, C. J. *et al.* (2013) Application of massively parallel sequencing to genetic diagnosis in multiplex families with idiopathic sensorineural hearing impairment PLoS One **8**, e57369 10.1371/journal.pone.0057369

44. Fromer, M., Pocklington, A. J., Kavanagh, D. H., Williams, H. J., Dwyer, S., Gormley, P. *et al.* (2014) De novo mutations in schizophrenia implicate synaptic networks Nature **506**, 179-184 10.1038/nature12929

45. Kim, S. J., Lee, S., Park, H. J., Kang, T. H., Sagong, B., Baek, J. I. *et al.* (2016) Genetic association of MYH genes with hereditary hearing loss in Korea Gene **591**, 177-182 10.1016/j.gene.2016.07.011

46. Song, M. H., Jung, J., Rim, J. H., Choi, H. J., Lee, H. J., Noh, B. *et al.* (2020) Genetic Inheritance of Late-Onset, Down-Sloping Hearing Loss and Its Implications for Auditory Rehabilitation Ear Hear **41**, 114-124 10.1097/AUD.0000000000000734

47. Wang, Z., Huang, C., Sun, Y., Lv, H., Zhang, M., andLi, X. (2019) Novel mutations associated with autosomal-dominant congenital cataract identified in Chinese families Exp Ther Med **18**, 2701-2710 10.3892/etm.2019.7865

48. Frohne, A., Koenighofer, M., Liu, D. T., Laccone, F., Neesen, J., Gstoettner, W. *et al.* (2021) High Prevalence of MYO6 Variants in an Austrian Patient Cohort With Autosomal Dominant Hereditary Hearing Loss Otol Neurotol **42**, e648-e657 10.1097/MAO.0000000000003076

49. Skiver, B. M., Patel, S. B., andBose, P. (2013) It is not always immune thrombocytopenia: a case of MYH9-related platelet disorder caused by a novel mutation Eur J Haematol **91**, 191-192 10.1111/ejh.12104

50. Schleinitz, N., Favier, R., Mazodier, K., Difeo, A., Ebbo, M., Veit, V. *et al.* (2006) [The MYH9 syndrome: report of a new case with a new mutation of the MYH9 gene] Rev Med Interne **27**, 783-786 10.1016/j.revmed.2006.07.012

51. Savoia, A., andPecci, A. (1993) MYH9-Related Disease In GeneReviews((R)), Adam MP, Everman DB, Mirzaa GM, Pagon RA, Wallace SE, Bean LJH, et al., eds. Seattle (WA)

52. Miyagawa, M., Naito, T., Nishio, S. Y., Kamatani, N., andUsami, S. (2013) Targeted exon sequencing successfully discovers rare causative genes and clarifies the molecular epidemiology of Japanese deafness patients PLoS One **8**, e71381 10.1371/journal.pone.0071381

53. Li, J., Wang, L., Wan, L., Lin, T., Zhao, W., Cui, H. *et al.* (2019) Mutational spectrum and novel candidate genes in Chinese children with sporadic steroid-resistant nephrotic syndrome Pediatr Res **85**, 816-821 10.1038/s41390-019-0321-z

54. Sloan-Heggen, C. M., Bierer, A. O., Shearer, A. E., Kolbe, D. L., Nishimura, C. J., Frees, K. L. *et al.* (2016) Comprehensive genetic testing in the clinical evaluation of 1119 patients with hearing loss Hum Genet **135**, 441-450 10.1007/s00439-016-1648-8

55. Ma, E. S., Wong, C. L., Shek, T. W., andHui, S. P. (2006) Hematologic and genetic characterization of an MYH9-related disorder in a Chinese family Haematologica **91**, 1002-1003, <https://www.ncbi.nlm.nih.gov/pubmed/16818291>

56. Pecci, A., Panza, E., De Rocco, D., Pujol-Moix, N., Girotto, G., Podda, L. *et al.* (2010) MYH9 related disease: four novel mutations of the tail domain of myosin-9 correlating with a mild clinical phenotype Eur J Haematol **84**, 291-297 10.1111/j.1600-0609.2009.01398.x

57. Ghemlas, I., Li, H., Zlateska, B., Klaassen, R., Fernandez, C. V., Yanofsky, R. A. *et al.* (2015) Improving diagnostic precision, care and syndrome definitions using comprehensive next-generation sequencing for the inherited bone marrow failure syndromes J Med Genet **52**, 575-584 10.1136/jmedgenet-2015-103270

58. Sanders, S. J., Murtha, M. T., Gupta, A. R., Murdoch, J. D., Raubeson, M. J., Willsey, A. J. *et al.* (2012) De novo mutations revealed by whole-exome sequencing are strongly associated with autism Nature **485**, 237-241 10.1038/nature10945

59. Wang, Y., Li, D., Xu, Y., Ma, L., Lu, Y., Wang, Z. *et al.* (2018) Functional Effects of SNPs in MYH9 and Risks of Nonsyndromic Orofacial Clefts J Dent Res **97**, 388-394 10.1177/0022034517743930

60. Provaznikova, D., Geierova, V., Kumstyrova, T., Kotlin, R., Mikulenkova, D., Zurkova, K. *et al.* (2009) Clinical manifestation and molecular genetic characterization of MYH9 disorders Platelets **20**, 289-296 10.1080/09537100902993022

61. Pitsava, G., Feldkamp, M. L., Pankratz, N., Lane, J., Kay, D. M., Conway, K. M. *et al.* (2021) Exome sequencing of child-parent trios with bladder exstrophy: Findings in 26 children Am J Med Genet A **185**, 3028-3041 10.1002/ajmg.a.62439

62. Rojas-Restrepo, J., Caballero-Oteyza, A., Huebscher, K., Haberstroh, H., Fliegauf, M., Keller, B. *et al.* (2021) Establishing the Molecular Diagnoses in a Cohort of 291 Patients With Predominantly Antibody Deficiency by Targeted Next-Generation Sequencing: Experience From a Monocentric Study Front Immunol **12**, 786516 10.3389/fimmu.2021.786516

63. Capalbo, A., Valero, R. A., Jimenez-Almazan, J., Pardo, P. M., Fabiani, M., Jimenez, D. *et al.* (2019) Optimizing clinical exome design and parallel gene-testing for recessive genetic conditions in preconception carrier screening: Translational research genomic data from 14,125 exomes PLoS Genet **15**, e1008409 10.1371/journal.pgen.1008409

64. de Smith, A. J., Lavoie, G., Walsh, K. M., Aujla, S., Evans, E., Hansen, H. M. *et al.* (2019) Predisposing germline mutations in high hyperdiploid acute lymphoblastic leukemia in children Genes Chromosomes Cancer **58**, 723-730 10.1002/gcc.22765

65. Boucher, S., Tai, F. W. J., Delmaghani, S., Lelli, A., Singh-Estivalet, A., Dupont, T. *et al.* (2020) Ultrarare heterozygous pathogenic variants of genes causing dominant forms of early-onset deafness underlie severe presbycusis Proc Natl Acad Sci U S A **117**, 31278-31289 10.1073/pnas.2010782117

66. Yamanouchi, J., Hato, T., Kunishima, S., Niiya, T., Nakamura, H., andYasukawa, M. (2015) A novel MYH9 mutation in a patient with MYH9 disorders and platelet size-specific effect of romiplostim on macrothrombocytopenia Ann Hematol **94**, 1599-1600 10.1007/s00277-015-2416-x

67. Burstein, D. S., Gaynor, J. W., Griffis, H., Ritter, A., Connor, M. J. O., Rossano, J. W. *et al.* (2021) Genetic variant burden and adverse outcomes in pediatric cardiomyopathy Pediatr Res **89**, 1470-1476 10.1038/s41390-020-1101-5

68. Ali, S., Ghosh, K., Daly, M. E., Hampshire, D. J., Makris, M., Ghosh, M. *et al.* (2016) Congenital macrothrombocytopenia is a heterogeneous disorder in India Haemophilia **22**, 570-582 10.1111/hae.12917
